# Revealing the role of cancer-associated fibroblast senescence in prognosis and immune landscape in pancreatic cancer

**DOI:** 10.1016/j.isci.2024.111612

**Published:** 2024-12-16

**Authors:** Luyao Liu, Hai Huang, Bin Cheng, Huaping Xie, Wang Peng, Haochen Cui, Jingwen Liang, Mengdie Cao, Yilei Yang, Wei Chen, Ronghua Wang, Yuchong Zhao

**Affiliations:** 1 Department of Gastroenterology and Hepatology, Tongji Hospital, Tongji Medical College, Huazhong University of Science and Technology, Wuhan 430030, China; 2 Department of Surgery, University of Pittsburgh School of Medicine, Pittsburgh, PA 15213, USA

**Keywords:** Microenvironment, Immune response, Cancer

## Abstract

Cancer-associated fibroblasts (CAFs) represent a major contributor to tumor growth. Cellular senescence is a state of cell-cycle arrest characterized by a pro-inflammatory phenotype. The potential impact of CAF senescence on tumor progression and the tumor microenvironment (TME) remains to be elucidated. Here, we systematically investigated the relationship between CAF senescence and the TME of pancreatic ductal adenocarcinoma (PDAC) based on multi-omics analysis and functional experiments. CAF senescence promotes tumor progression *in vitro* and *in vivo* and contributes to the formation of immunosuppressive TME. A CAF-senescence-related risk score was developed to predict overall survival, immune landscape, and treatment sensitivity in patients with PDAC. Further experiments revealed that plasminogen activator urokinase (PLAU) derived from senescent CAFs (SCAFs) promoted PDAC progression and was involved in immunosuppression. Together, these findings suggested that CAF senescence was correlated with tumor progression, and the CAF-senescence-based machine learning model could potentially predict prognosis in patients with PDAC.

## Highlights

• Cancer-associated fibroblasts (CAFs) exhibit high senescence levels in PDAC
• Senescent CAFs (SCAFs) promote PDAC progression and immunosuppression
• The CSscore is correlated with the prognosis and therapy sensitivity of patients with PDAC
• SCAF-derived PLAU promotes PDAC progression *in vitro* and *in vivo*

## Graphical abstract

**Figure:**
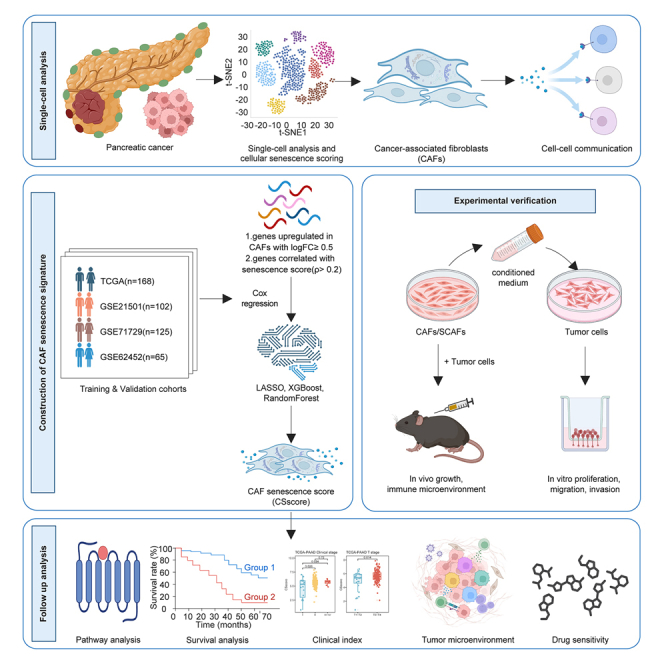

## Introduction

Pancreatic ductal adenocarcinoma (PDAC) remains one of the most lethal malignancies globally, with a dismal five-year survival rate of merely 13%.^1^ This poor prognosis is largely attributed to its highly aggressive nature, late-stage diagnosis, and resistance to conventional therapies such as chemotherapy and radiotherapy.^2^ Recent research has highlighted the critical role of the tumor microenvironment (TME) in PDAC progression.^3^ The TME of PDAC is a complex ecosystem comprising cancer cells, cancer-associated fibroblasts (CAFs), extracellular matrix (ECM) components, endothelial cells, and immune cells.^4^ Among these, CAFs have garnered significant attention due to their critical roles in PDAC progression.^5^ CAFs produce abundant ECM components surrounding the tumor, creating a mechanical barrier that impedes drug penetration and immune cell infiltration.^6^ Furthermore, CAFs contribute to immunosuppression by releasing factors such as phospholipase A2 group IIA (PLA2G2A), macrophage colony stimulating factor 1 (MCSF), and stromal cell-derived factor 1α (SDF-1α) to impair CD8^+^ T cell anti-tumor activity, promote anti-inflammatory macrophages phenotypes, and recruit myeloid-derived suppressor cells (MDSCs).^7^^,^^8^^,^^9^

Cellular senescence, recently included in the “hallmark of cancer,” has emerged as a critical regulator of tumor biology.^10^ Senescence is characterized by an irreversible cell-cycle arrest and a distinct bioactive and pro-inflammatory secretome, termed the senescence-associated secretory phenotypes (SASPs).^11^ Senescent cells are present in tumors at various stages and can be triggered by multiple stressors, including DNA damage, oncogenic activation, oxidative stress, nutrient depletion, and cancer therapies.^12^ Senescence can also occur in CAFs, altering their influence on tumor progression.^13^ In breast cancer models, senescent CAFs (SCAFs) have been shown to promote tumor growth by modifying the ECM components and suppressing natural killer (NK) cell infiltration.^14^ SCAFs contribute to the stemness of breast cancer cells by providing aspartate and proline to support tumor growth.^15^ SCAFs have also been implicated in therapy resistance. Upon radiotherapy or chemotherapy, inflammatory CAFs undergo senescence, secreting cytokines, and ECM constituents that support tumor metastasis to mediate therapy resistance in rectal cancer.^16^ Clearance of SCAFs has synergized with radiotherapy in breast cancer models.^17^ Given the abundance of CAFs in the TME of PDAC, understanding the role of SCAFs in PDAC biology is crucial for unraveling tumor progression mechanisms and identifying novel therapeutic targets.

In this study, we systematically explored senescence status in various cellular components of the TME of PDAC and found CAFs exhibiting a high level of senescence. We then curated a comprehensive panel of CAF-senescence-related genes and employed machine-learning algorithms to identify genes significantly associated with PDAC patient prognosis. Based on the refined gene sets, we established a CAF-senescence-score (CSscore) and investigated its relationship with prognosis, functional enrichment pathways, immune landscape, and therapy sensitivity of PDAC. Furthermore, we validated the role of SCAF-secreted plasminogen activator urokinase (PLAU) in tumor progression and the cultivation of immunosuppressive TME of PDAC through *in vitro* and *in vivo* experiments. In conclusion, our study demonstrated the prognostic values and tumor-promoting roles of CAF senescence, providing new insights into the mechanisms of how CAF senescence contributed to PDAC progression.

## Results

### Cancer-associated fibroblasts exhibit high senescence levels in the tumor microenvironment of pancreatic ductal adenocarcinoma

**Figure 1:**
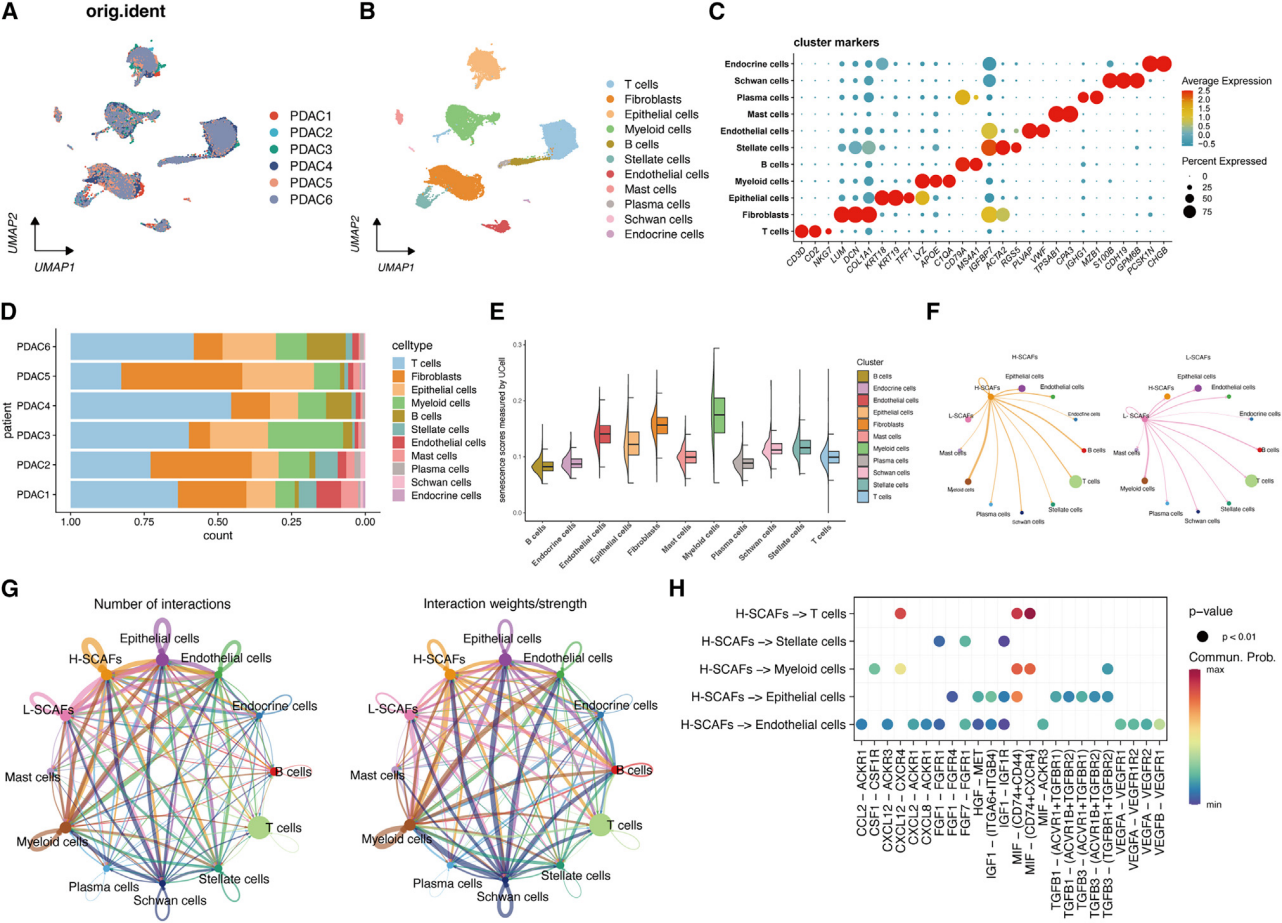
The senescence status of CAFs in the TME of PDAC (A) UMAP plot showing the profile of PDAC samples. (B) Visualization of 11 cell types. (C) The dot plot of cell markers used for cluster annotation. (D) The proportions of all cell types. (E) Half violin plot of the cellular senescence score across cell clusters. (F and G) Circle plots of the cellular interaction weights and number of interactions between H-SCAFs, L-SCAFs, and other cells. g. Bubble plot of significant ligand-receptor pairs contributing to the signaling sending from H-SCAFs to other cell types.

To investigate cellular senescence within the TME of PDAC, we analyzed a published single-cell RNA sequencing (scRNA-seq) dataset containing six PDAC patient samples.^18^ After quality control, we performed clustering analysis on 31,362 cells using the uniform manifold approximation and projection (UMAP) method. The resulting UMAP plot showed a uniform distribution of cells across samples, indicating successful batch effect removal (Figure 1A). Based on established marker gene expression, we identified 11 distinct cell populations (Figures 1B and 1C), including five immune cell types (plasma cells, mast cells, B cells, myeloid cells, and T cells) and six non-immune cell types (CAFs, epithelial cells, endothelial cells, stellate cells, endocrine cells, and Schwann cells). Analysis of the relative proportions of each cell type across the six PDAC samples revealed substantial intratumoral heterogeneity, particularly in T cells and CAFs (Figure 1D). To assess cellular senescence, we employed the SenMayo senescence gene set (Table S1)^19^ for UCell enrichment analysis.^20^ This analysis showed that among non-immune cells, CAFs exhibited the highest senescence scores, second only to myeloid cells in general (Figure 1E). Given the crucial role of CAFs in shaping the TME and their abundance in PDAC stroma, we focused on CAFs for further investigation.

Senescent cells are characterized by senescence-associated secretory phenotypes (SASPs), comprising pro-inflammatory cytokines and chemokines, growth regulators, and angiogenic factors.^21^ To investigate potential SASP-mediated interactions between SCAFs and other cells in the TME, we stratified CAFs into high-senescent (H-SCAFs) and low-senescent (L-SCAFs) subsets based on the median senescence score. Cell-cell communication analysis revealed that H-SCAFs exhibited a higher degree of interactions compared to L-SCAFs, particularly with myeloid cells, T cells, and endothelial cells (Figures 1F and 1G; Figures S1A and S1B). Notably, H-SCAFs secreted macrophage migration inhibitory factor (MIF) and C-X-C motif chemokine ligand 12 (CXCL12) to interact with T cells via the MIF-CD74/CD44, MIF-CD74/CXCR4, and CXCL12-CXCR4 signaling axes (Figure 1H). Additionally, H-SCAFs delivered vascular endothelial growth factor (VEGF) to endothelial cells via VEGFA-VEGFR1, VEGFA-VEGFR1R2, VEGFA-VEGFR2, and VEGFB-VEGFR1 signaling nodes (Figure 1H). These data suggested that SCAF-derived SASP factors, including CXCL12, MIF, and VEGF, potentially mediated immunomodulation and angiogenesis in the PDAC TME.^22^^,^^23^^,^^24^

### Senescent cancer-associated fibroblasts promote pancreatic ductal adenocarcinoma progression and impair anti-tumor immunity

**Figure 2:**
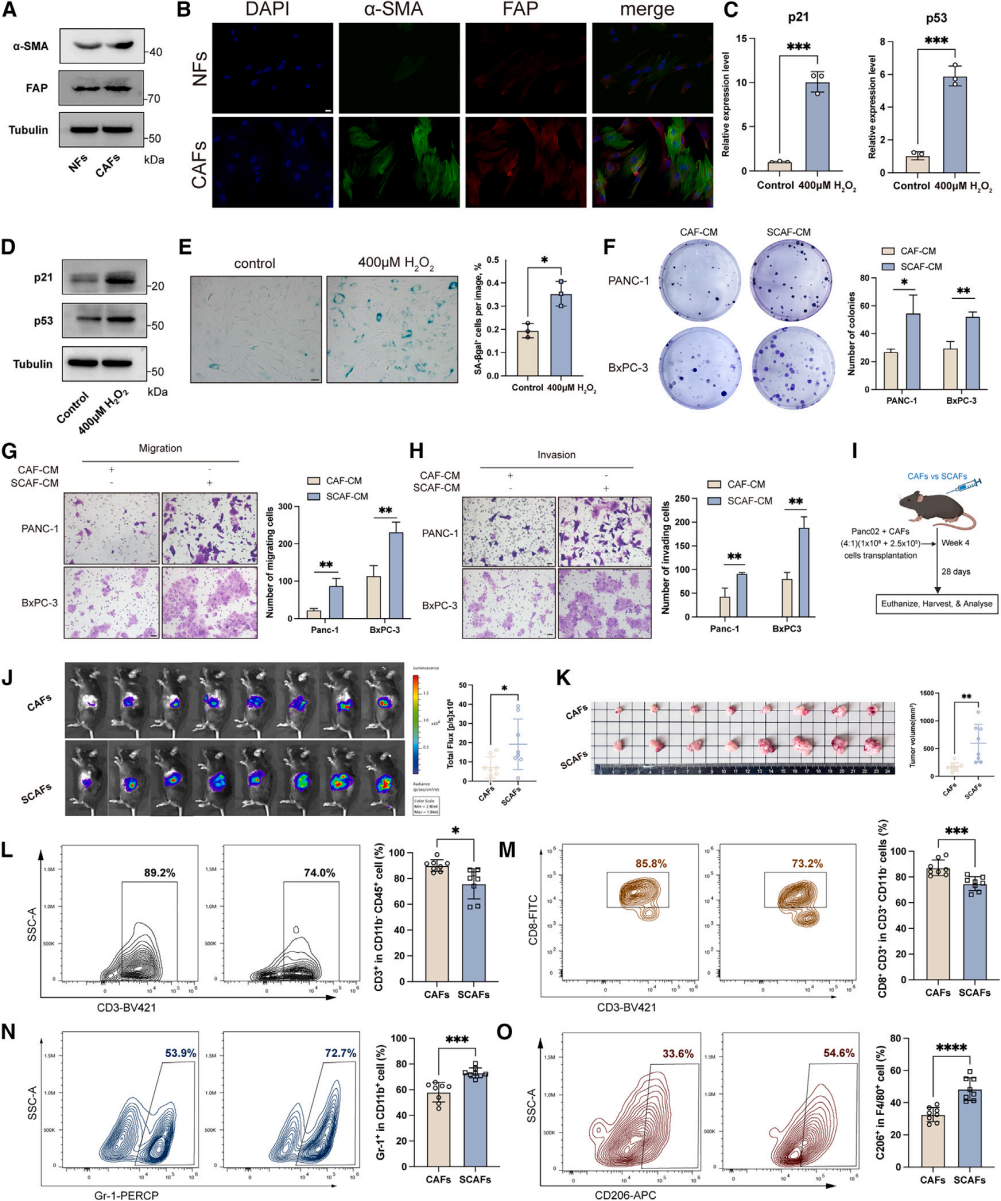
SCAFs promote PDAC progression and impair anti-tumor immunity (A) Protein expression for α-SMA and FAP in human CAFs and NFs was measured by western blot. (B) Representative images of immunofluorescence for α-SMA and FAP in human CAFs and NFs. Scale bar, 20 μm. (C) Relative mRNA expression of p21 and p53 in human CAFs treated with 400 μM H_2_O_2_ was measured by qRT-PCR. (D) Protein expression of p53 and p21 in human CAFs treated with 400 μM H_2_O_2_ was measured by western blot. (E) SA-β-Gal staining of human CAFs treated with 400 μM H_2_O_2_. Scale bar, 20 μm. (F) The proliferation ability of the indicated cells was measured by colony formation assay. (G and H) The migration and invasion abilities of the indicated cells were measured by Transwell assay. Scale bar, 20 μm. (I) The flow of the experimental design. (J) Bioluminescence images showing orthotopically transplanted PDAC tumors. (K) Photographs and volumes of tumors in C57BL/6 mice. (L and M) Representative flow cytometry images and statistical analysis of CD3^+^ (L) and CD8^+^ T cells (M) in tumors in different groups. (N and O) Representative flow cytometry images and statistical analysis of MDSCs (N) and M2 macrophage (O) in tumors in different groups. Data are represented as mean ± SD. ∗*p* < 0.05, ∗∗*p* < 0.01, ∗∗∗*p* < 0.001.

To investigate the impact of SCAFs on PDAC progression, we first isolated CAFs and normal fibroblasts (NFs) from human PDAC tissues and adjacent normal tissues. The western blot and immunofluorescence analyses revealed that CAFs showed higher expression levels of α-smooth muscle actin (α-SMA) and fibroblast activation protein (FAP) compared to NFs (Figures 2A and 2B). Next, we induced CAF senescence using hydrogen peroxide (H_2_O_2_) (400 μM) as previously reported to trigger oxidative stress-induced cellular senescence.^25^ Increased mRNA and protein levels of senescence markers p21 and p53 (Figures 2C and 2D), and elevated senescence-associated β-galactosidase (SA-β-Gal) activity were detected in H_2_O_2_-induced SCAFs (Figure 2E). Besides, the expression levels of genes encoding ECM modulatory factors, cytokines, and insulin-like growth factor (IGF) family members were upregulated in the H_2_O_2_-induced SCAFs (Figure S2A), all of which were previously identified as elevated in SCAFs from human PDAC.^26^

The results of colony formation and CCK-8 assays showed that PANC-1 and BxPC-3 cells treated with SCAF-conditioned media (SCAF-CM) exhibited significantly enhanced proliferation ability compared to those treated with CAF-CM (Figures 2F and S2B). Moreover, the results of Transwell assay revealed that tumor cells treated with SCAF-CM showed increased migration and invasion capacities (Figures 2G and 2H).

To further demonstrate the pro-tumorigenic role of CAF senescence in PDAC progression *in vivo*, we isolated mouse CAFs from KPC (*Kras*^*LSL-G12D/+*^*; Trp53*^*LSL-R172H/+*^*; Pdx1-Cre*) mice (Figure S2C) and confirmed their identity by immunofluorescence analyses (Figure S2D). Mouse CAFs were induced to the senescence phenotype, showing elevated mRNA and protein expression levels of p21 and p53, and increased SA-β-Gal activity (Figures S2E–S2G). In addition, we observed the upregulation of ECM-related factors and immunomodulatory factors in the H_2_O_2_-induced mouse SCAFs (Figure S2H), consistent with the expression profiles identified in reported mouse PDAC SCAFs.^26^ We then co-injected these cells with Panc02 cells into immunocompetent C57BL/6J mice to construct an orthotopic xenograft mouse model (Figure 2I). Bioluminescence imaging revealed that the group of Panc02 cells co-injected with SCAFs exhibited significantly increased tumor burden and tumor volume (Figures 2J and 2K). Flow cytometry analyses were employed to further elucidate the differences in the TME shaped by SCAFs and CAFs, and reduced infiltrations of CD3^+^ T cells and CD8^+^ T cells (Figures 2L and 2M) but increased infiltrations of MDSCs and M2 macrophages were observed in the SCAF group (Figures 2L–2O). Similarly, immunohistochemical staining revealed significant increased staining of Ki67^+^ cells, CD206^+^ cells, and Gr-1^+^ cells, and marked reductions in CD8^+^ cells in the SCAF group (Figure S2I). These results collectively suggested that SCAFs contributed to cancer proliferation and the cultivation of immunosuppressive TME in PDAC.

### A gene signature based on cancer-associated fibroblast senescence improves prognostic prediction in pancreatic ductal adenocarcinoma

**Figure 3:**
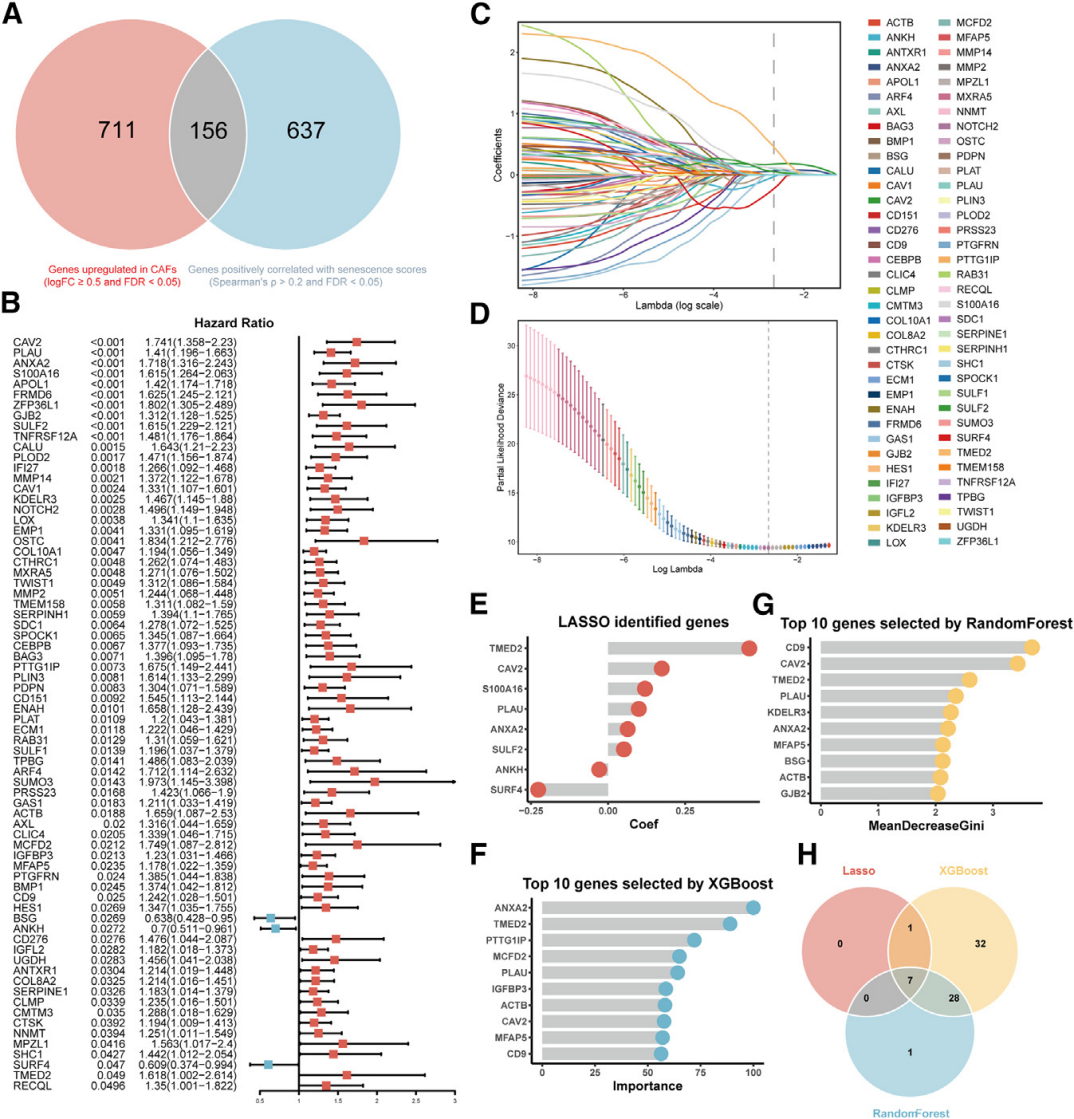
Development of the risk signature based on machine-learning (A) Venn diagram of the overlap of genes elevated in CAFs and genes positively correlated with the cellular senescence scores in CAFs. (B) 72 genes with *p* < 0.05 filtered by univariate Cox survival analysis in the CAF senescence signature. (C) LASSO coefficient profiles of the 72 genes. (D) 10-fold cross-validation to select tuning parameters. (E) LASSO coefficient profiles of the 8 selected genes. (F and G) Top 10 genes selected by XGBoost (F) and RandomForest (G), respectively. (H) Venn diagram of the intersection of prognostic genes selected by three machine-learning approaches.

Given the tumor-promoting roles of SCAFs, we next assessed the prognostic values of a CAF-senescence-related gene signature. We conducted differential expressed gene (DEG) analysis using the scRNA-seq dataset.^18^ Genes expressed in CAFs with a higher level (logFC≥ 0.5, pct≥ 0.5, and FDR< 0.05) compared to all other cell types were identified as CAF-upregulated genes (Table S2). Genes positively correlated with the senescence scores in CAFs were considered senescence-related genes (Spearman ρ > 0.2 and FDR< 0.05). With intersection, 156 genes were obtained and utilized to construct a gene signature reflecting SCAF characteristics in PDAC (Figure 3A; Table S3). Functional enrichment analyses of Gene Ontology (GO) terms and the Reactome pathway revealed that the CAF senescence signature was enriched by genes associated with ECM-related processes, cell adhesion, and intercellular interactions (Figure S3A).

To investigate the impact of CAF senescence on PDAC progression, we calculated a SCAF index (SCAF enrichment scores/CAF enrichment scores) using the single-sample gene set enrichment analysis (ssGSEA). Kaplan-Meier analysis showed that a higher SCAF index was associated with shorter survival time in patients with PDAC (Figure S3B). In addition, a higher SCAF index was positively correlated with advanced clinical stages, higher T stages, and worse histological grades in patients with PDAC (Figure S3C), suggesting that CAF senescence was associated with poor prognosis.

**Figure 4:**
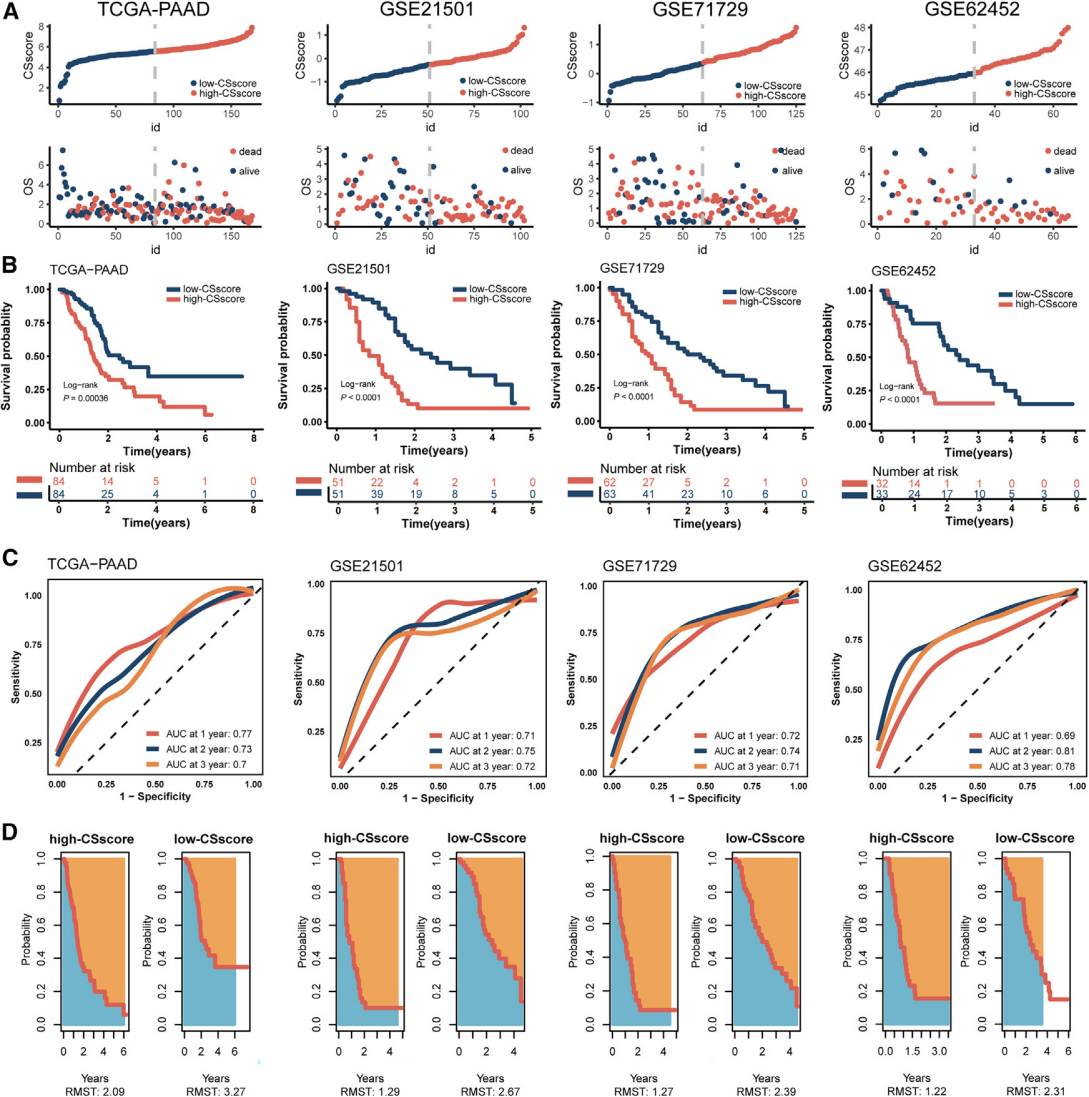
Stratified survival analysis of the CSscore signature (A) Distribution of the CSscore according to the survival status and time in TCGA-PAAD, GSE21501, GSE71729, and GSE62452 cohorts. (B) Kaplan-Meier survival analysis of OS for patients with high- and low-CSscore grouped by the median value in 4 cohorts. (C) Time-dependent ROC curves of 1-year, 2-year, and 3-year OS in 4 cohorts. (D) Restricted mean survival time between high- and low-CSscore groups grouped by the median value in 4 cohorts.

To optimize the CAF senescence signature for survival prognostication, we performed univariate Cox regression analysis, identifying 72 genes significantly associated with prognosis (Figure 3B). We then employed three machine-learning algorithms (LASSO, XGBoost, and RandomForest) to select candidate prognostic genes, yielding 8 (Figures 3C–3E), 68 (Figures 3F and S3D), and 36 genes (Figures 3G, S3E, and S3F), respectively. The intersection of these results identified 7 hub genes (Figure 3H), which were used to construct the CAF-senescence-score (CSscore) using Cox proportional hazards regression in the TCGA-PAAD training set. The CSscore for each patient was calculated based on the Cox coefficients and log2 (FPKM+1) transformed expression values of the 7 genes. We stratified patients into high- and low-CSscore groups based on the median value across four cohorts (TCGA-PAAD and three independent validation cohorts: GSE21501,^27^ GSE71729,^28^ and GSE62452,^29^) (Figure 4A). Kaplan-Meier analysis demonstrated that elevated CSscore were significantly associated with worse overall survival (OS) across all four cohorts (Figure 4B). Receiver Operating Characteristic (ROC) curve analysis validated the prognostic utility of the CSscore, with Area Under Curve (AUC) values of 0.77, 0.73, and 0.70 for 1-, 2-, and 3-year OS in TCGA-PAAD; 0.71, 0.75, and 0.72 in GSE21501; 0.72, 0.74, and 0.71 in GSE71729; and 0.69, 0.81, and 0.78 in GSE62452, respectively (Figure 4C). Furthermore, restricted mean survival time (RMST) analysis revealed higher mean survival times and lower mortality rates in the low-CSscore groups compared to their high-CSscore counterparts, with differences observed at 6 years in TCGA-PAAD, 4 years in GSE21501 and GSE71729, and 3 years in GSE62452 (Figure 4D).

### Establishing the CAF-senescence-score as a robust prognostic signature

**Figure 5:**
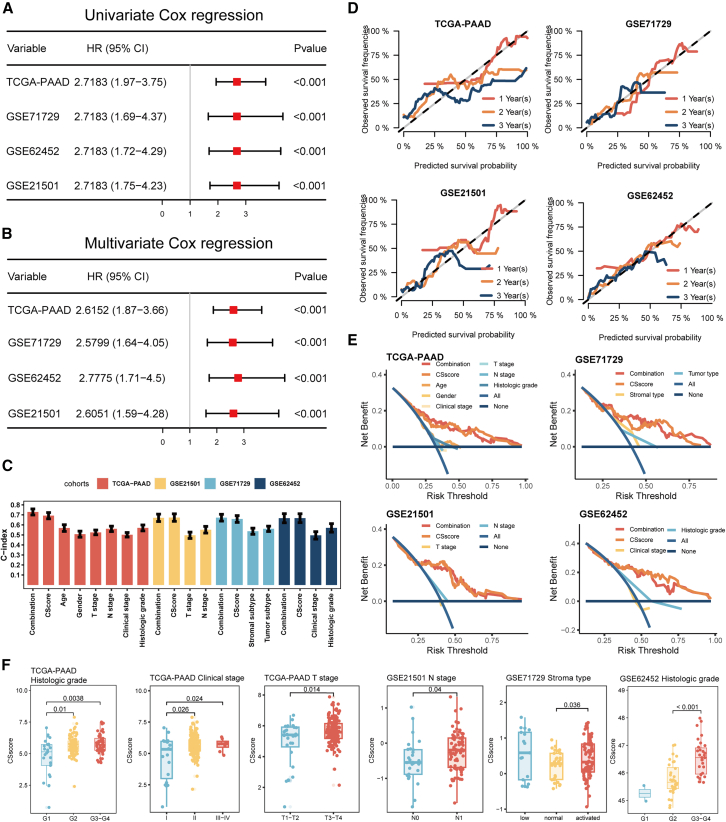
Assessment of the CSscore prediction model (A) Forest plot of univariate Cox regression analysis of the CSscore in TCGA and GEO datasets. (B) Forest plot of multivariate Cox regression analysis of the CSscore in TCGA and GEO datasets. (C) The C-index of the CSscore, other clinical factors, and the combination signature in TCGA and GEO datasets. (D) The 1-year, 2-year, and 3-year calibration curves of the CSscore in TCGA and GEO datasets. (E) The decision curves of the CSscore, other clinical factors, and the combination signature in TCGA and GEO datasets. (F) Difference analysis of the distribution of the CSscore in different T stages, N stages, clinical stages, histological grades, and molecular subtypes of patients with PDAC.

We assessed the prognostic performance of clinicopathologic factors in PDAC using the C-index. The CSscore emerged as an independent predictor of OS in both univariate and multivariate Cox regression analyses (Figures 5A and 5B; Figures S4A–S4D), demonstrating superior prognostic utility compared to established clinicopathologic parameters including age, N stage, and histologic grade (Figure 5C). Furthermore, a prognostic signature integrating the CSscore with age, gender, T stage, N stage, clinical stage, and histologic grade exhibited enhanced predictive capability relative to any single clinicopathologic parameter (Figure 5C). Calibration plots validated the robust predictive accuracy of the CSscore across risk strata (Figure 5D). Decision curve analysis revealed a substantial clinical net benefit of applying the CSscore (Figure 5E). In addition, elevated CSscores showed significant positive correlations with advanced histologic grades in the TCGA-PAAD and GSE62452 cohort, aggressive molecular subtypes in GSE71729 cohort, higher T stages and clinical stages in the TCGA-PAAD cohort, and higher N stages in GSE21501 cohort (Figure 5F). These findings collectively established the CSscore as a robust prognostic biomarker, with elevated CSscore associated with worse clinical outcomes in PDAC.

### Potential biological mechanisms related to the CAF-senescence-score

**Figure 6:**
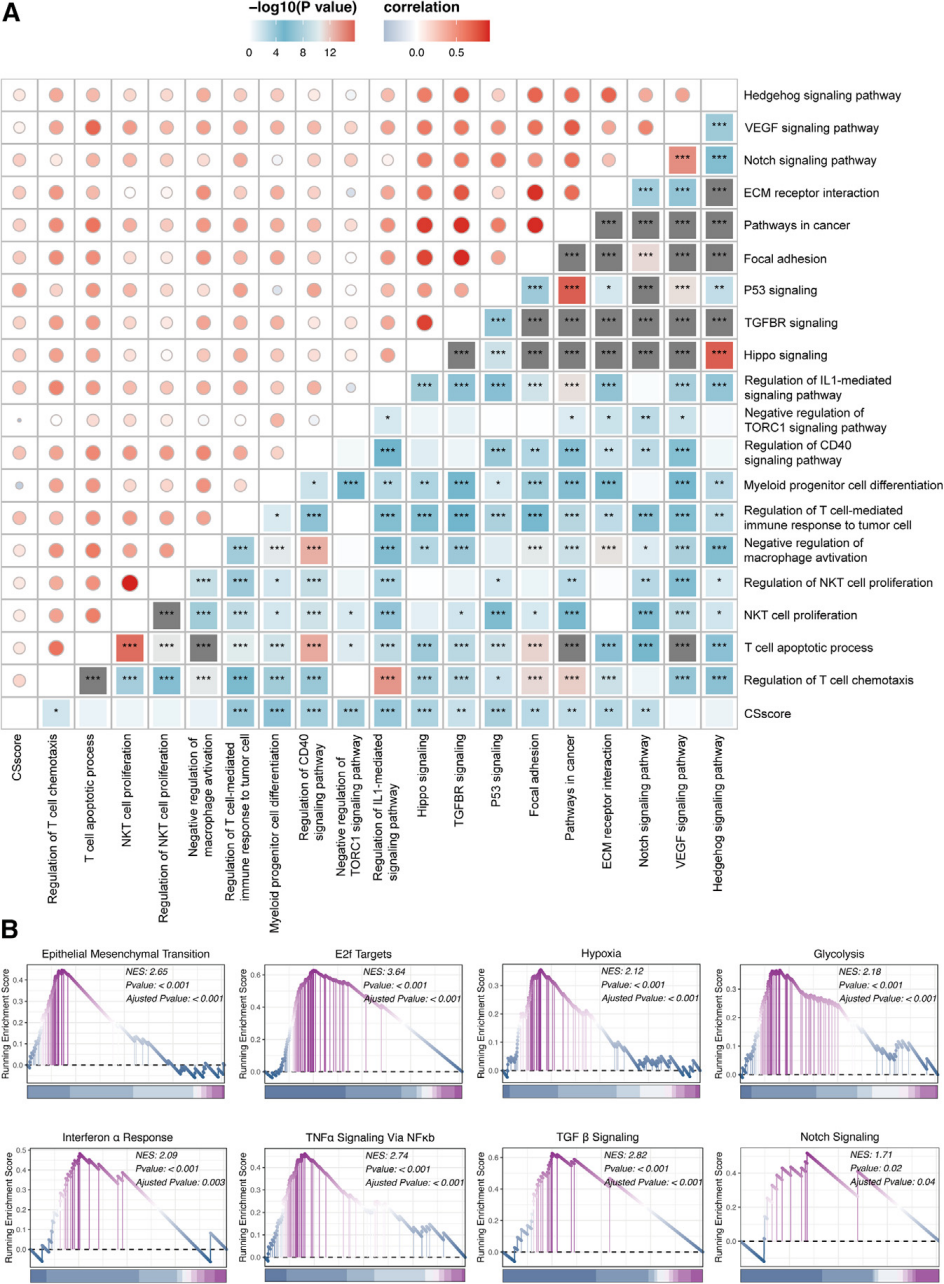
Functional annotation of the CSscore in the TCGA-PAAD cohort (A) Heatmap displaying the correlation between the CSscore and tumor-associated pathways, and immune-related pathways based on GSVA of GO and KEGG terms using Spearman’s test. (B) GSEA of Hallmark pathways for the CSscore in the TCGA-PAAD dataset. ∗*p* < 0.05, ∗∗*p* < 0.01, ∗∗∗*p* < 0.001.

To elucidate the biological mechanisms associated with the CSscore, we performed functional enrichment analysis of GO and Kyoto Encyclopedia of Genes and Genomes (KEGG) terms using the Gene Set Variation Analysis (GSVA). The results found positive correlations between elevated CSscores and enrichment of pathways regarding T cell chemotaxis, myeloid progenitor differentiation, CD40 signaling, Notch signaling, Hippo signaling, and focal adhesion (Figure 6A). Concordantly, the Gene Set Enrichment Analysis (GSEA) of the Hallmark gene sets found that epithelial-mesenchymal transition (EMT), E2F targets, glycolysis, and TNFα signaling via NF-κB were more enriched in the high-CSscore subset (Figure 6B).

### Correlation of the CAF-senescence-score with immune infiltration

**Figure 7:**
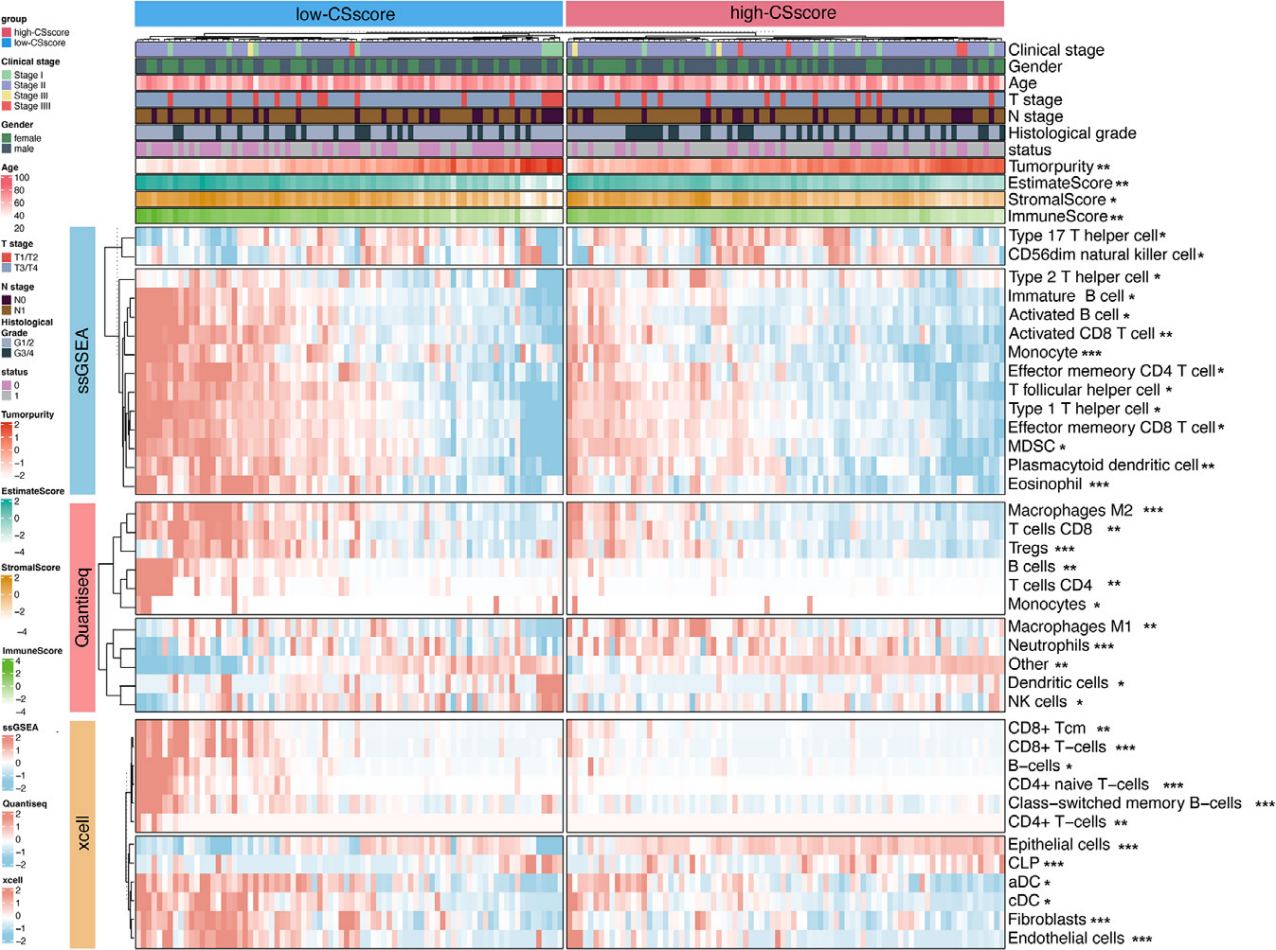
Immune-related characteristics of the CSscore signature in the TCGA-PAAD dataset Difference of immune infiltration between the low- and high-CSscore groups. The results were shown in the form of a complex heatmap, in which the abundance of immune cell infiltration measured by the ssGSEA, quanTIseq, and xCell algorithms between the two groups was presented in the form of a heatmap. The immune score, stromal score, ESTIMATE score, and tumor purity predicted by the ESTIMATE algorithm were displayed in the form of a bar chart. ∗*p* < 0.05, ∗∗*p* < 0.01, ∗∗∗*p* < 0.001.

We further analyzed the associations between the CSscore signature and immune cell infiltration (Figure 7). The ESTIMATE algorithm revealed significantly elevated immune scores in the low-CSscore subset, alongside increased tumor purity in the high-CSscore group. The infiltration of CD8^+^ and CD4^+^ T lymphocytes has been implicated in promoting anti-tumor immunity in PDAC.^30^^,^^31^ NK cells, which recognize and eliminate tumor cells, are often functionally impaired in PDAC.^32^ The ssGSEA found the enrichment of activated CD8^+^ T cells, effector memory CD8^+^ T cells, and type 1 helper T cells in the low-CSscore group. Conversely, type 2 and type 17 helper T cells, reported previously to be correlated with poor prognosis in PDAC,^33^^,^^34^ were enriched in the high-CSscore subset. The QuanTIseq and xCell analyses further supported these findings, revealing significant enrichment of CD4^+^ T cells, CD8^+^ T cells, and NK cells in the low-CSscore group. Neutrophils, involved in promoting PDAC progression, showed enrichment in the high-CSscore subset based on the quanTIseq results.

Differences in immune cell infiltration levels could be attributed to varying tumor aggressiveness. To investigate whether the observed differences in immune cell infiltration between the high- and low-CSscore groups were influenced by tumor aggressiveness, we compared the compositional differences of various clinical characteristics between the two groups. The results revealed no significant differences in the compositions of genders, clinical stages, T stages, N stages, and histological grades between the two groups (Figure S5), suggesting that the observed differences in immune landscape were associated with CAF senescence.

### Role of the CAF-senescence-score in therapy sensitivity

To investigate the relationship between the CSscore signature and immunotherapy response, we employed the Tracking Tumor Immunophenotype (TIP) tool to assess anti-tumor immune responses^35^ (Figure S6). Our analysis revealed significant negative correlations between CSscore and several anti-tumor immune processes, including "Priming and activation (step 3)", "Recognition of cancer cells by T cells (step 6)", and "Dendritic cell recruiting (step 4)." Conversely, increased CSscores were positively correlated with immunosuppressive activities such as "MDSCs recruiting (step 4)", "Neutrophil recruiting", and "Regulatory T cell recruiting." These results suggested that high CSscores were associated with dysregulated anti-cancer immune responses.

**Figure 8:**
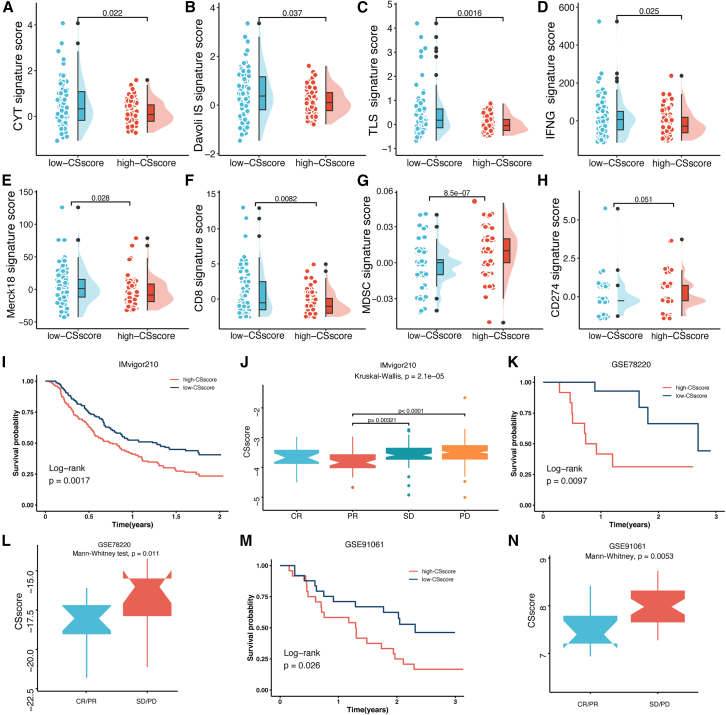
Relation of the CSscore with immunotherapy-associated biomarkers (A–H) Boxplot displaying the enrichment scores of CYT (A), Davoli IS (B), TLS (C), IFNγ (D), Merck18 (E), CD8 (F), MDSC (G), and CD274 (H) gene signatures between the low- and high-CSscore groups. (I) Kaplan-Meier survival analysis of the CSscore regarding OS in the IMvigor210 cohort. (J) Difference analysis of the CSscore in different immunotherapy responses in the IMvigor210 cohort. (K) Kaplan-Meier survival analysis of the CSscore regarding OS in the GSE78220 cohort. (L) Difference analysis of the CSscore in different immunotherapy responses in the GSE78220 cohort. (M) Kaplan-Meier survival analysis of the CSscore regarding OS in the GSE91061 cohort. (N) Difference analysis of the CSscore in different immunotherapy responses in the GSE91061 cohort. ∗*p* < 0.05, ∗∗*p* < 0.01, ∗∗∗*p* < 0.001.

Further analysis revealed that the low-CSscore group exhibited significantly higher enrichment scores of gene signatures associated with better immunotherapy response, including Cytotoxic activity (CYT),^36^ Davoli immune signature (Davoli IS),^37^ tertiary lymphoid structures (TLS),^38^ IFN-γ (IFNG), Merck18 (T-cell-inflamed signature), and CD8 signature scores (Figures 8A–8F). In contrast, the high-CSscore group showed elevated levels of MDSC and CD274 signature scores, indicators of poorer immunotherapy response (Figures 8G and 8H). Moreover, survival analysis in the IMvigor210 cohort^39^ revealed shorter survival time and worse responses to anti-PD-1 immunotherapy in patients with higher CSscores (Figures 8I and 8J). Similar findings were observed in immunotherapy cohorts of non-small cell lung cancer (GSE78220,^40^) and melanoma (GSE91061,^41^) (Figures 8K–8N). These findings suggested that patients with a low-CSscore might benefit more from immunotherapy.

Additionally, we computed half-maximal inhibitory concentration (IC50) values for various anti-tumor drugs in patients with PDAC. The low-CSscore cohort exhibited significantly lower IC50 values for gemcitabine, cisplatin, irinotecan, olaparib, oxaliplatin, and nilotinib (Figure S7), suggesting that patients with PDAC with low CSscores might be more sensitive to these therapeutic agents.

### Senescent cancer-associated fibroblasts-derived plasminogen activator urokinase promotes pancreatic ductal adenocarcinoma progression *in vitro* and *in vivo*

**Figure 9:**
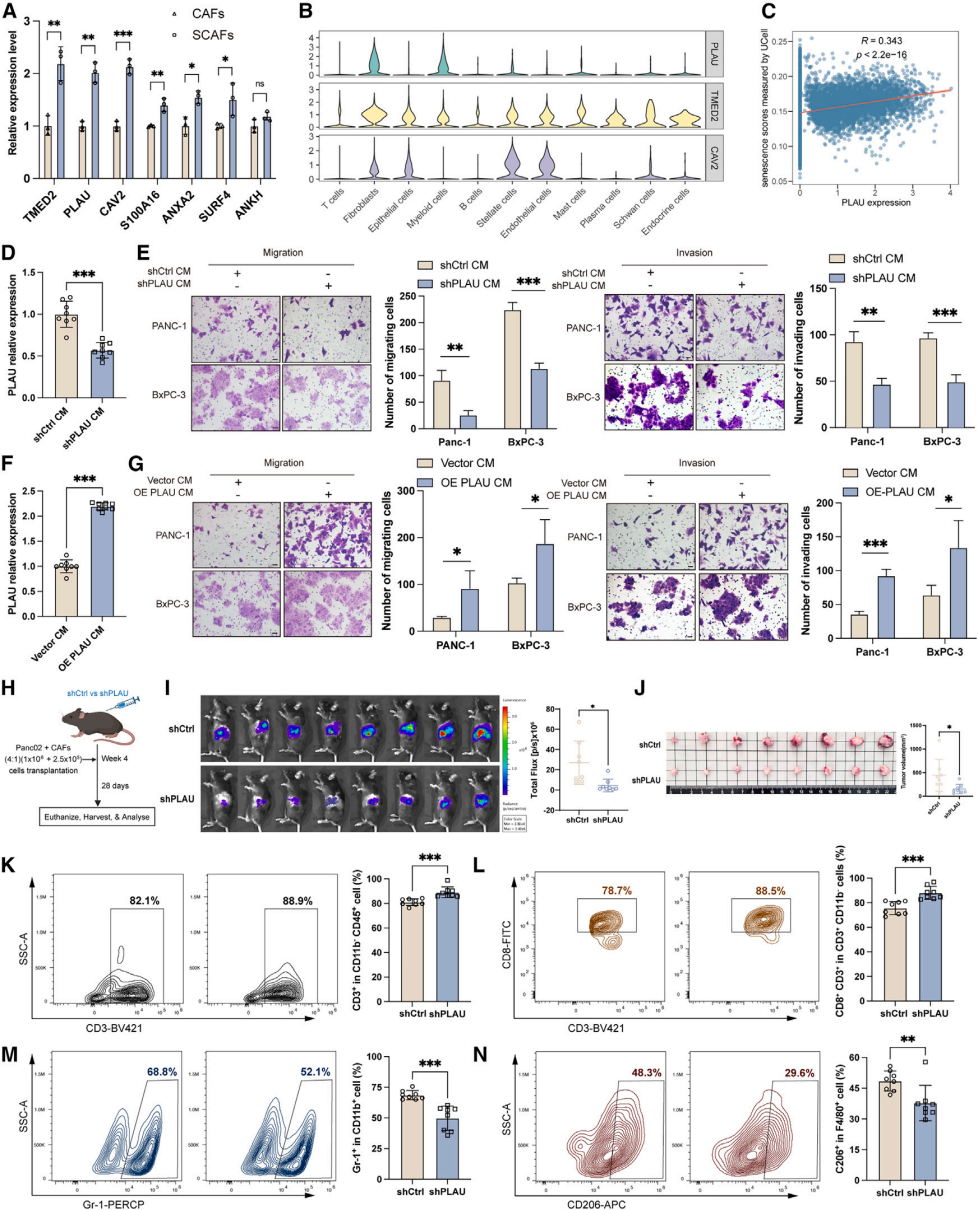
PLAU derived from SCAFs promotes PDAC progression *in vitro* and *in vivo* (A) Relative mRNA expression of genes in human CAFs treated with 400 μM H_2_O_2_ was measured by qRT-PCR. (B) The mRNA expression levels of genes in various cell types in the TME of PDAC. (C) The relationship between PLAU expression and UCell senescence score in CAFs. (D) PLAU knockdown in human SCAFs was verified by ELISA. (E) The migration and invasion abilities of the indicated cells were assessed by Transwell assay. Scale bar, 20 μm. (F) PLAU overexpression in human CAFs was verified by ELISA. (G) The migration and invasion abilities of the indicated cells were assessed by Transwell assay. Scale bar, 20 μm. (H) The flow of the experimental design. (I) Bioluminescence images showing orthotopically transplanted PDAC tumors. (J) Photographs and volumes of tumors. (K and L) Representative flow cytometry images and statistical analysis of CD3^+^ (K) and CD8^+^ T cells (L) in tumors in different groups. (M and N) Representative flow cytometry images and statistical analysis of MDSCs (M) and M2 macrophage (N) in tumors in different groups. Data are represented as mean ± SD. ∗*p* < 0.05, ∗∗*p* < 0.01, ∗∗∗*p* < 0.001.

To validate the CSscore signature, we evaluated the expression levels of the seven hub genes in SCAFs. The expression levels of transmembrane p24 trafficking protein 2 (TMED2), caveolin 2 (CAV2), and plasminogen activator urokinase (PLAU) were significantly upregulated by more than 2-fold in SCAFs compared to controls (Figure 9A). TMED2 plays a crucial role in vesicular protein trafficking between the endoplasmic reticulum and the Golgi apparatus.^42^ CAV2, a major component of caveolae, is involved in the negative regulation of the TGF-β signaling pathway.^43^ PLAU encodes a secreted serine protease involved in ECM remodeling and cell migration.^44^^,^^45^ While CAV2 and TMED2 exhibited widespread expression in both tumor cells and stromal cells, PLAU demonstrated a relatively specific expression pattern in CAFs and myeloid cells (Figure 9B). In addition, the scRNA-seq analysis found that PLAU expression was strongly correlated with the cellular senescence scores in CAFs (R = 0.343, *p* < 0.001) (Figures 9C, S8A, and S8B). The DEG analysis between H-SCAFs and L-SCAFs in the scRNA-seq dataset revealed that while CAV2 and TMED2 showed no significant difference, PLAU was significantly upregulated in H-SCAFs (logFC = 0.773) (Figure S8C). Given these findings and the secreted nature, we selected PLAU for further exploration.

PLAU protein levels were elevated in SCAFs as well (Figure S8D). We downregulated PLAU in SCAFs by transfecting shPLAU plasmids and verified its knockdown efficiency by Enzyme-linked immunosorbent assay (ELISA) (Figure 9D) and western blot (Figure S8E). The results of colony formation and CCK-8 assays revealed that PDAC cells treated with CM from PLAU-knockdown SCAFs (PLAU-knockdown SCAF-CM) exhibited decreased proliferation capacity (Figures S8F and S8G). The results of the Transwell assay revealed that PDAC cells treated with PLAU-knockdown SCAF-CM showed attenuated migration and invasion abilities (Figure 9E). Conversely, we upregulated PLAU in CAFs (via plasmids) and validated its overexpression by ELISA and western blot (Figures 9F and S8H). The results of colony formation, CCK-8, and Transwell assay indicated that PDAC cells treated with CM from PLAU-overexpressing CAFs (PLAU-overexpressing CAF-CM) showed increased proliferative, migratory, and invasive capacities (Figures S8I and S8J; Figure 9G).

To investigate the role of PLAU *in vivo*, we constructed stable Plau knockdown in SCAFs from KPC mice via lentiviral shRNA transduction (Figures S8K and S8L). We then constructed the orthotopic pancreatic tumor model by co-implanting these Plau-knockdown SCAFs with Panc02 cells (Figure 9H). Mice bearing Plau-knockdown SCAFs exhibited significantly reduced tumor burden and volume compared to the control group (Figures 9I and 9J). Flow cytometry analysis revealed that Plau-knockdown SCAFs promoted the infiltration of CD3^+^ and CD8^+^ T cells in the TME and reduced the infiltration of MDSCs and M2 macrophages (Figures 9K–9N). The results of immunohistochemical staining were consistent with these findings, showing decreased staining of Ki67^+^, CD206^+^, and Gr-1^+^ cells, and increased staining of CD8^+^ cells in the Plau-knockdown SCAF group (Figure S8M). In addition, the TIP tool was applied to analyze the relationship between PLAU expression and anti-tumor immune response. The activity of "Macrophage recruiting", "MDSC recruiting", and "Treg cell recruiting" was positively related to PLAU expression (Figure S9A), which implied the immunosuppressive role of PLAU. Furthermore, higher PLAU expression was found to be associated with increased levels of M2 macrophage markers (CD206 and CD163) (Figure S9B). Correlation analysis also revealed a significantly positive correlation between PLAU expression and expression levels of CD206 and CD163 (Figure S9C). By applying the “TIP” tool, we found a negative correlation between PLAU expression and the abundance of CD4^+^ naive cells, although this correlation did not reach statistical significance (Figure S9D). In summary, these findings suggested that SCAF-derived PLAU promoted tumor proliferation and the formation of immunosuppressive TME in PDAC.

### Senescent cancer-associated fibroblasts-derived plasminogen activator urokinase promotes pancreatic cancer cell migration via the Notch1 signaling pathway

Next, we aimed to investigate the mechanism by which SCAF-derived PLAU promoted pancreatic cancer progression. Prior studies have shown that the Notch signaling pathway can induce EMT and promote tumor invasion.^46^ We performed GSEA analysis in the TCGA-PAAD cohort based on the transcriptional expression of PLAU and found enrichment of the Notch pathway in the group with high PLAU expression (Figures S10A and S10B). The correlation analysis showed positive associations between PLAU expression and key components of the Notch pathway, including Notch1, Notch1 intracellular domain (NICD), hes family bHLH transcription factor 1 (Hes1), and hes related family bHLH transcription factor with YRPW motif 1 (Hey1) (Figures S10C–S10E). Western blot confirmed that PDAC cells treated with PLAU-knockdown SCAF-CM showed decreased expression of Notch1, NICD, Hes1, and Hey1 (Figure S10F). Conversely, PDAC cells treated with PLAU-overexpressing CAF-CM exhibited increased expression of these proteins (Figure S10G).

To further elucidate the role of Notch1 signaling in PLAU-mediated cancer progression, we constructed PDAC cells with stable overexpression and knockdown of Notch1 (via lentiviral transduction). The efficiency of overexpression and knockdown of Notch1 in PANC-1 and BxPC-3 cell lines were validated by real-time PCR (Figures S11A and S11B), western blot (Figures S11C and S11D), and immunofluorescence staining (Figures S11E and S11F). When PDAC cells were treated with PLAU-knockdown SCAF-CM, the migration and invasion capacity of PDAC cells were partially rescued by Notch1 overexpression (Figures S11G and S11H). Western blot analysis revealed that Notch1 overexpression also restored the expression of NICD, Hes1, and Hey1 in these PDAC cells (Figure S11I). Conversely, when PDAC cells were treated with PLAU-overexpressing CAF-CM, the migration and invasion capacity of PDAC cells were inhibited by Notch1 knockdown (Figures S11J and S11K). In addition, Notch1 knockdown also decreased the expression of NICD, Hes1, and Hey1 in these PDAC cells (Figure S11L). These findings collectively suggested that SCAF-secreted PLAU promoted PDAC cell migration and invasion by activating the Notch1 signaling pathway.

## Discussion

Our study presented several important findings that advanced the understanding of CAF senescence in PDAC. Firstly, we developed and validated the CSscore as an independent prognostic factor for OS in patients with PDAC. Second, we explored the associations between the CSscore and clinicopathological parameters, immune infiltration, functional pathways, and therapy sensitivity. Finally, through multi-omics analysis and functional experiments, we identified PLAU derived from SCAFs as a critical mediator promoting PDAC progression and immunosuppression.

Cellular senescence plays complex roles in tumor biology. While senescence can initially suppress tumorigenesis in premalignant cells, it may also contribute to therapy resistance and tumor progression, particularly through SASPs. Senescence can occur in stromal cells, especially CAFs.^13^ Through scRNA-seq analysis, we revealed that CAFs exhibited high senescence levels in the TME of PDAC. The triggers for CAF senescence in PDAC have not been fully elucidated. SCAFs are often observed in closer proximity to tumor cells compared to non-senescent CAFs, suggesting that tumor cells play a critical role in initiating CAF senescence.^26^ Studies have demonstrated that tumor cells induce CAF senescence through secreted factors such as CXCL1 in ovarian cancer^47^ and interleukin-1α (IL-1α) in rectal cancer.^48^ Additionally, the characteristic desmoplasia of the PDAC TME, which results in abnormal vasculature, hypoxia, and nutrient depletion, may trigger senescence in CAFs.^4^ Cancer therapies such as chemotherapy and radiotherapy can also induce CAF senescence.^16^^,^^48^

SCAFs have been identified as a subpopulation of myofibroblastic CAFs that accumulate as tumors progress from pancreatic intraepithelial neoplasia (PanIN) to developed PDAC.^26^^,^^49^ Depletion of SCAFs has been shown to alleviate immunosuppression, enhance effector T cell function, and improve the efficacy of chemotherapy and immunotherapy in PDAC.^26^^,^^49^ Consistent with these findings, our study supported the role of SCAFs in immunosuppression in PDAC. The cell communication analysis revealed that H-SCAFs exhibited higher cellular communication levels with other cell types, particularly immune cells. H-SCAFs were identified as a prominent source of MIF, a cytokine involved in promoting M2 macrophage polarization, MDSC recruitment, and T cell exhaustion.^23^^,^^50^^,^^51^ Further flowmetry analysis showed that SCAFs increased the infiltration of M2 macrophages and MDSCs while reducing CD3^+^ and CD8^+^ T cell infiltration. Furthermore, our study established the CSscore signature based on CAF senescence and demonstrated its value in predicting prognosis and therapy sensitivity in patients with PDAC. Moreover, we found the SCAF-derived PLAU promoted tumor proliferation and the cultivation of immunosuppressive TME of PDAC, providing new insights into the mechanisms of how SCAFs promoted PDAC progression. In addition, CAF senescence is also observed in other solid tumors. In breast cancer, SCAFs promote tumor progression by inhibiting NK cell infiltration and killing capacity through ECM remodeling.^14^ In colorectal cancer, chemotherapy-induced SCAFs enhance cancer aggressiveness and chemoresistance through increased transforming growth factor β (TGF-β) secretion.^16^ These findings suggest that CAF-senescence-related signature has the potential to predict the prognosis and therapy sensitivity of various solid tumors. Future studies can focus on constructing a pan-cancer CAF senescence signature to assess its value in the survival prognostication and prediction of therapy sensitivity.

The CSscore consisted of seven CAF-senescence-related genes: TMED2, PLAU, CAV2, S100A16, ANXA2, SURF4, and ANKH. Among these, PLAU was a secreted factor significantly upregulated in SCAFs and its expression was strongly correlated with the UCell senescence scores in CAFs. Furthermore, PLAU showed a relatively specific expression pattern in CAFs and myeloid cells. Therefore, PLAU was selected for *in vitro* and *in vivo* experiments. Previous studies have implicated PLAU in immunosuppression using bioinformatic analysis and found that the pharmacological inhibition of PLAU reduced tumor metastases in an immunodeficient mouse model of PDAC.^52^ Our study revealed PLAU’s role in tumor progression specifically in the context of SCAFs. We found that PLAU knockdown in SCAFs decreased PDAC cell proliferation and invasion. Moreover, using an immunocompetent orthotopic xenograft mouse model, we found that SCAF-derived PLAU promoted the infiltration of MDSCs and M2 macrophages while inhibiting effector T cell infiltration. Interestingly, our TIP analysis did not show a significant correlation between PLAU expression and CD8^+^ effector T cell infiltration. This result might be attributed to the typically low levels of CD8^+^ effector T cells in the TME of PDAC, which might result in low expression of genes in the CD8^+^ effector T cell signature, potentially affecting the accuracy of our analysis. Previous studies have found that high expression of PLAU in cancer cells promoted their migration and invasion abilities by activating the ERK and PI3K/Akt signaling pathways.^53^^,^^54^ In our study, we found that PLAU derived from SCAFs enhanced PDAC cell migration and invasion by activating the Notch1 signaling pathway. This finding provided new insights into the understanding of SCAF-mediated tumor-promoting mechanisms.

An interesting point of our findings was the differences between the SenMayo gene set and our CAF-senescence-related gene signature. CAFs and myeloid cells exhibited high enrichment scores for the SenMayo gene set. PLAU, a hub gene of our CAF senescence signature, was found to be specifically expressed in CAFs and myeloid cells. However, the genes comprising these two signatures were markedly different. The SenMayo gene set consists of a broad spectrum of SASPs, transmembrane, and intracellular proteins known to be enriched in senescent cells.^19^ In contrast, our CAF senescence signature was constructed by intersecting CAF-upregulated genes with senescence-related genes, reflecting characteristics of senescence specifically in CAFs within the PDAC TME. Unlike the enrichment of chemokine factors in the SenMayo gene set, the CAF senescence signature was prominently enriched by genes regarding ECM-related processes. These differences highlighted the unique aspects of CAF senescence within the TME of PDAC.

Our Ucell enrichment analysis revealed that myeloid cells had the highest senescence scores in the TME of PDAC. While the role of senescent myeloid cells in PDAC remains to be elucidated, studies in other cancers have revealed that they can mediate immunosuppression and drive tumor progression. For instance, in KRAS-driven lung cancer, senescent macrophages accumulate in tumor tissues as tumors progress and depletion of senescent macrophages ameliorates tumorigenesis by enhancing immunosurveillance by effector T cells.^55^^,^^56^ In breast cancer, senescent dendritic cells induced by γδ regulatory T cells suppress effector T cell function by inhibiting Type 1 helper T cell and Type 17 helper T cell differentiation and promoting regulatory T cell development.^57^ In prostate cancer, senescent neutrophils contribute to tumor proliferation and T cell suppression.^58^ These findings warrant further investigation of senescent myeloid cells in PDAC.

In conclusion, the CSscore developed in this study represented a practical prognostic signature capable of predicting survival outcomes and therapy sensitivity of patients with PDAC. Our functional experiments validated that SCAF-derived PLAU contributed to tumor progression and the formation of immunosuppressive TME of PDAC. We also revealed that SCAF-derived PLAU promoted PDAC cell migration and invasion by activating the Notch1 signaling axis. These findings not only extended the understanding of CAF senescence in PDAC progression but also presented new targets for therapeutic intervention.

### Limitations of the study

The present study has several potential limitations. First, the CSscore signature was acquired from retrospective data based on public datasets. Consequently, the clinical utility of this signature requires validation through prospective studies with larger, independent patient cohorts. Second, due to the high intratumoral heterogeneity of CAFs and potential sampling bias, additional single-cell transcriptomic analyses from different patients with PDAC are needed to further validate our findings. Moreover, we have not tested whether SCAFs have any effects on other immune cells, including CD4^+^ T cells and regulatory T cells. Further investigations are required to evaluate the impacts of SCAFs on other immune cell recruitment. Additionally, we did not assess the effects of SCAFs on immune cell functions *in vitro*. Further studies will be required to test the consequences of SCAFs on immune cell functions, such as macrophage polarization, and MDSC migration.

## Resource availability

### Lead contact

Further information and requests for resources should be directed to and will be fulfilled by the lead contact, Yuchong Zhao (zhaoyuchongtj@163.com).

### Materials availability

The materials will be available upon request.

### Data and code availability

• This article analyzes existing, publicly available data. These accession numbers for the datasets are listed in the key resources table.
• All codes used for the bioinformatics analysis in this study were derived from existing algorithms, as listed in the key resources table. The code was reposited at the following link: (https://github.com/llyao-work/CS_PDAC).
• Any additional information required to reanalyze the data reported in this article is available from the lead contact upon reasonable request.

## Acknowledgments

This research is supported by the 10.13039/501100001809National Natural Science Foundation of China (No. 82372867) and the 10.13039/501100003819Hubei Provincial Natural Science Foundation of China (No. 2024AFB829). The authors would like to thank the help of the Department of Gastroenterology and Hepatology, Tongji Hospital, Tongji Medical College, Huazhong University of Science and Technology.

## Author contributions

Conceptualization, B.C. and Y.Z.; formal analysis, L.L.; methodology, L.L., H.H., W.P., and M.C.; investigation, L.L., H.H., H.C., W.P., J.L., Y.Y., W.C., and M.C.; resources, B.C. and Y.Z.; writing – original draft, L.L., B.C., and Y.Z.; writing – review and editing, H.C., H.X., and R.W.; supervision, B.C. and Y.Z.; funding acquisition, B.C. and Y.Z.

## Declaration of interests

The authors declare no competing interests.

## STAR★Methods

### Key resources table

**Table:** 

REAGENT or RESOURCE	SOURCE	IDENTIFIER
**Antibodies**		

FITC anti-mouse CD8a	BD Horizon	Cat#553030; RRID:AB_394568
PE anti-mouse F4/80	BD Horizon	Cat#565410; RRID:AB_2687527
PerCP anti-mouse Ly6C/Ly6G	BD Horizon	Cat#552093; RRID:AB_394334
APC-Cy7 anti-mouse CD45	BD Horizon	Cat#557659; RRID:AB_396774
APC anti-mouse CD206	BD Horizon	Cat#565250; RRID:AB_2739133
BV421 anti-mouse CD3e	BD Horizon	Cat#562600; RRID:AB_11153670
BV510 anti-mouse CD11b	BD Horizon	Cat#562950; RRID:AB_2737913r
Purified Rat Anti-Mouse CD16/CD32 (Mouse BD Fc Block)	BD Horizon	Cat#553141; RRID:AB_394656
Anti-a-SMA/ACTA2 Antibody (Clone#1A4)	BOSTER	Cat#BM0002; RRID:AB_2811044
Vimentin Antibody	ABGENT	Cat#AX10005
FAP monoclonal antibody (M01), clone 1E5	Abnova	Cat#H00002191-M01; RRID:AB_538711
NOTCH1 Polyclonal antibody	Proteintech	Cat#20687-1-ap; RRID:AB_10700012
Cleaved Notch1 Rabbit mAb	Cell Signaling Technology	Cat#4147; RRID:AB_2153348
HEY1 Polyclonal antibody	Proteintech	Cat#19929-1-AP; RRID:AB_10646438
P53 Monoclonal antibody	Proteintech	Cat#60283-2-Ig; RRID:AB_2881401
Alpha Tubulin Monoclonal antibody	Proteintech	Cat#66031-1-Ig; RRID:AB_11042766
HES1 Rabbit pAb	Abclonal	Cat#A11718; RRID:AB_2758714
PLAU Rabbit pAb	Abclonal	Cat#A2181; RRID:AB_2764199
Waf1/Cip1/CDKN1A p21 Antibody (F-5)	Santa cruz	Cat#sc-6246; RRID:AB_628073
Ki-67 (D3B5) Rabbit mAb	Cell Signaling Technology	Cat#9129; RRID:AB_2687446
Anti-CD8 alpha antibody	Abcam	Cat#ab209775; RRID:AB_2860566
CD206/MRC1 (E6T5J) XP® Rabbit mAb	Cell Signaling Technology	Cat#24595; RRID:AB_2892682
Ly-6G/Ly-6C Monoclonal Antibody	Invitrogen	Cat#14-5931-82; RRID:AB_467730
Alexa Fluor® 488-conjugated Goat Anti-Rabbit IgG (H + L)	Servicebio	Cat#Gb25303; RRID:AB_2910224
Alexa Fluor® 488-conjugated Goat Anti-Mouse IgG (H + L)	Servicebio	Cat#GB25301; RRID:AB_2904018
Cy3-Conjugated Goat Anti-Rabbit IgG (H + L)	Servicebio	Cat#GB21303; RRID:AB_2861435
Cy3 Conjugated Goat Anti-mouse IgG (H + L)	Servicebio	Cat#GB21301; RRID:AB_2923552

**Bacterial and virus strains**		

Lentiviral vector	Genechem Corporation (Shanghai, China)	N/A

**Biological samples**		

PDAC tissues and adjacent non-tumorous tissues	Tongji Hospital, Huazhong University of Science and Technology	N/A

**Chemicals, peptides, and recombinant proteins**		

DMEM media	GIBCO	Cat#11965092
Penicillin-Streptomycin (10,000 U/mL)	NCM Biotech	Cat#C100C5
Phosphate Buffered Saline	Servicebio	Cat#G4202
Fetal Bovine Serum	GIBCO	Cat#MT35010CV
Matrigel	Corning	Cat#356234
DAPI Staining Reagent (Ready to Use)	Servicebio	Cat#G1012
Triton X-100	Servicebio	Cat#G3068-100ML
RIPA Buffer (Enhanced)	Wuhan Promotor Biological CO., LTD	Cat#B1025
Phosphatase Inhibitor Cocktail I (100× in DMSO)	MedChemExpress	Cat#HY-K0021
Phenylmethylsulfonyl fluoride (PMSF)	MedChemExpress	Cat#HY-B0496
5× SDS-PAGE Loading Buffer (Reduced)	Servicebio	Cat#G2013
SuperKine™ West Femto Maximum Sensitivity Substrate	Abbkine	Cat#BMU102
Trometamol(Tris)	GENVIEW	Cat#BT350
Glycine	GENVIEW	Cat#FG149
Skim Milk Powder	Biosharp	Cat#BS102
SDS	GENVIEW	Cat#GS286
Bovine Serum Albumin, fraction V	GENVIEW	Cat#FA016
Prestained Protein Marker (10-250kDa)	BOSTER	Cat#AR1210
Tween 20	Ding Guo	Cat#DH358-4
Pierce™ BCA Protein Assay Kits	Thermo Fisher Scientific	Cat#23225
Cell Counting Kit-8	YEASEN	Cat#40203ES80
TRIzol™ Reagent	Invitrogen	Cat#15596026CN
3% hydrogen peroxide solution	BOSTER	Cat#AR1108
Hanks' Balanced Salt Solution	Servicebio	Cat#G4203-500ML
Collagenase Type I	GIBCO	Cat#17100017
Trypsin-EDTA (0.25%)	YEASEN	Cat#40127ES60
MycoBlue Mycoplasma Detector	Vazyme	Cat#D101-01
D-Luciferin potassium	MCE	Cat#HY-12591B
Lipofectamine 3000	Invitrogen	Cat#L3000015
Polybrene Infection/Transfection Reagent	Sigma-Aldrich	Cat#TR-1003-G
Puromycin	MCE	Cat#HY-B1743A
ECL detection kit	Abbkine	Cat#BMU102-EN
4% paraformaldehyde	YEASEN	Cat#60536ES76
Tris-EDTA buffer	Proteintech	Cat#PR30002
DAB kit	Proteintech	Cat#PR30010

**Critical commercial assays**		

HiScript II Q RT SuperMix for qPCR	Vazyme	Cat#R222-01
ChamQ Universal SYBR qPCR Master Mix	Vazyme	Cat#Q711-02
Human uPA/Urokinase/PLAU ELISA Kit	BOSTER	Cat#EK0535
mouse uPA/Urokinase/PLAU ELISA Kit	Hengyuan Biological Technology (Shanghai, China)	Cat#HB514-Mu
Senescence β-Galactosidase Staining Kit	Beyotime	Cat#C0602
Tumor Dissociation Kit, mouse	Miltenyi Biotec	Cat#130-096-730

**Deposited data**		

TCGA-PAAD RNA-seq data	UCSC Xena	https://xenabrowser.net/
Gene expression microarray data	Jeran K Stratford et al.^27^	GEO: GSE21501
Gene expression microarray data	Richard A Moffitt et al.^28^	GEO: GSE71729
Gene expression microarray data	Shouhui Yang et al.^29^	GEO: GSE62452
Gene expression microarray data	Dung-Tsa Chen et al.^59^	GEO: GSE57495
Bulk RNA-seq data	Willy Hugo et al.^40^	GEO: GSE78220
Bulk RNA-seq data	Nadeem Riaz et al.^41^	GEO: GSE91061
Bulk RNA-seq data from IMvigor210 clinical trial	Banchereau et al.^39^	https://researchpub.gene.com/IMvigor210CoreBiologies
Single-cell RNA-seq data	Kai Chen et al.^18^	GEO: GSE212966
Gene expression microarray data	Francesco Puleo et al.^60^	ArrayExpress: E-MTAB-6134
Bulk RNA-seq data	ICGC database^61^	ICGC: PACA-CA

**Experimental models: Cell lines**		

Human: BxPC-3	ATCC	Cat#CRL-1687
Human: PANC-1	Cell Resource Center, Institute of Biochemistry and Cell Biology at the Chinese Academy of Science (Shanghai, China)	Cat#SCSP-535
Human: 293T	Cell Resource Center, Institute of Biochemistry and Cell Biology at the Chinese Academy of Science (Shanghai, China)	Cat#SCSP-502
Mouse: Panc02	BOSTER	Cat#CX0396

**Experimental models: Organisms/strains**		

C57BL/6	Hubei Biont Biological Technology Co., Ltd	N/A

**Oligonucleotides**		

Primers for virus-based RNAi, see Table S4	This paper	N/A
Primers for PLAU expression, see Table S5	This paper	N/A
Primers for qRT-PCR, see Table S6	This paper	N/A

**Software and algorithms**		

GraphPad Prism version 10.2 for Mac	GraphPad Software	https://www.graphpad.com/
Flowjo software v10	BD	https://www.flowjo.com/
R (v4.2.2)	R CRAN	https://cran.r-project.org/
Seurat V4	Yuhan Hao et al.^62^	https://satijalab.org/seurat/
affy	Laurent Gautier et al.^63^	https://bioconductor.org/packages/release/bioc/html/affy.html
Harmony	Ilya Korsunsky et al.^64^	https://portals.broadinstitute.org/harmony/
UCell	Massimo Andreatta et al.^20^	https://github.com/carmonalab/UCell
irGSEA	Chuiqin Fan et al.^65^	https://github.com/chuiqin/irGSEA
GSVA	Sonja Hänzelmann et al.^66^	https://github.com/rcastelo/GSVA
caret	Kuhn, Max et al.^67^	https://cran.r-project.org/web/packages/caret/index.html
glmnet	Friedman J et al.^68^	https://cran.r-project.org/web/packages/glmnet/index.html
randomForest	CRAN	https://cran.r-project.org/web/packages/randomForest/index.html
clusterProfiler	Tianzhi Wu et al.^69^	https://github.com/YuLab-SMU/clusterProfiler?tab=readme-ov-file
DESeq2	Michael I Love et al.^70^	https://bioconductor.org/packages/release/bioc/html/DESeq2.html
CellChat	Suoqin Jin et al.^71^	https://github.com/sqjin/CellChat
IOBR	Dongqiang Zeng et al.^72^	https://github.com/IOBR/
easier	Óscar Lapuente-Santana et al.^73^	https://github.com/olapuentesantana/easier
oncoPredict	Danielle Maeser et al.^74^	https://github.com/maese005/oncoPredict

**Other**		

PVDF paper	Merck millipore	Cat#IPVH00010
Original code used in this study	This paper	https://github.com/llyao-work/CS_PDAC

### Experimental model and study participant details

#### Primary PDAC specimens and isolation of primary fibroblasts

The use of human specimens in this study was approved by the Ethics Committee of Tongji Hospital, Huazhong University of Science and Technology (HUST), Wuhan, China (approval no. TJ-IRB202404100). Human PDAC tissues and adjacent non-tumorous tissues were obtained from three patients with PDAC undergoing surgery at the Department of Biliary-Pancreatic Surgery or the Department of Gastroenterology and Hepatology, Tongji Hospital, HUST. The patients or their guardians provided informed consent. None of the patients had received radiotherapy or chemotherapy before surgery. Patient information, including gender, can be found in Table S7. The tissue samples involved in this study are all derived from Asian Han patients with PDAC.

Mouse CAFs were isolated from the KPC (*Kras*^*LSL-G12D/+*^*; Trp53*^*LSL-R172H/+*^*; Pdx1-Cre*) mice with palpable tumors (>150 days old). Human CAFs and normal fibroblasts (NFs) were isolated from the collected PDAC specimens and adjacent normal pancreas tissues, respectively. All tissues were processed within 30 min of collection and kept on ice. The protocol of fibroblast isolation was modified from previously described methods.^75^^,^^76^ Briefly, after washing with Hanks' Balanced Salt Solution (Servicebio), tissues were cut into 1–2 mm^3^ pieces. The minced tissues were then dissociated in DMEM containing collagenase I (1 mg/mL, GIBCO) for 30 min at 37°C, and then plated in 6-well plates with DMEM (GIBCO) containing 15% FBS and 1% penicillin-streptomycin to allow attachment. Fibroblasts grew out of tumor fragments for 1–2 weeks. Upon reaching 80% confluence, cells were passaged using trypsin-EDTA and replated at a 1:3 ratio. To purify fibroblasts, cells were allowed to attach for 20 min in serum-free DMEM before changing the medium. Fibroblast purity was validated by immunofluorescence and western blot analysis for α-SMA and FAP, and their characteristic elongated morphology with cytoplasmic extensions. The antibodies used were anti-α-SMA antibody (BOSTER) and anti-FAP antibody (ABclonal). CAFs were used at passage numbers less than 10.

#### Cell lines

Human pancreatic cancer cell line BxPC-3 was obtained from the American Type Culture Collection (ATCC, Manassas, VA, USA). Human pancreatic cancer cell line PANC-1 and human embryonic kidney cell line 293-T were purchased from the Cell Resource Center, Institute of Biochemistry and Cell Biology at the Chinese Academy of Science (Shanghai, China). Mouse pancreatic cancer cell line Panc02 was acquired from Boster Biological Technology, Wuhan, China. Human CAFs and mouse CAFs were isolated from human PDAC samples and KPC mice PDAC samples, respectively. Cells were maintained in DMEM (GIBCO) comprising 10% FBS (GIBCO) and 1% penicillin-streptomycin at 37°C and 5% CO_2_. All cell lines used in this study were confirmed negative for mycoplasma using the MycoBlue Mycoplasma Detector (Vazyme).

#### Animal studies

All animal experiments were performed in accordance with the NIH Guide for the Care and Use of Laboratory Animals and approved by the Institutional Review Board of Tongji Hospital, HUST, Wuhan, China (approval no. TJH-202312031). Six-week-old male C57BL/6 mice were obtained from Hubei Biont Biological Technology Co., Ltd and housed under pathogen-free conditions. Mice were randomly allocated to experimental groups (*n* = 8 per group). For the mouse orthotopic model of pancreatic cancer, luciferase-labeled Panc02 cells (1× 10^6^) were mixed with CAFs (2.5× 10^5^) derived from PDAC tissues of KPC mice and injected into the tail of the pancreas in mice. Bioluminescence was assessed 3 weeks post-injection, 5 min after administering 100 μL of potassium D-luciferin salt (30 mg/mL in PBS) via intraperitoneal injection. Four weeks post-surgery, the mice were euthanized. Tumor volume was calculated using the formula: volume = XY^2^/2, where X represents the longest diameter, and Y represents the shortest of two perpendicular diameters.

### Method details

#### Data collection and processing

Raw bulk transcriptome counts, normalized and log2 transformed RNA-seq FPKM data, and clinical information in pancreatic cancer cohort in The Cancer Genome Atlas (TCGA) were sourced from the UCSC Xena database (https://xenabrowser.net/). The transcriptome expression data (GSE57495,^59^ GSE21501,^27^ GSE71729,^28^ GSE62452,^29^ GSE78220,^40^ and GSE91061,^41^) and the scRNA-seq data (GSE212966,^18^) were downloaded from the Gene Expression Omnibus (GEO) database (http://www.ncbi.nlm.nih.gov/geo/). The IMvigor210 dataset was obtained from https://researchpub.gene.com/IMvigor210CoreBiologies.^39^ The E-MTAB-6134 cohort^60^ was downloaded from the ArrayExpress database (https://www.ebi.ac.uk/biostudies/arrayexpress) and the PACA-CA dataset was acquired from the International Cancer Genome Consortium database (http://dcc.icgc.org/).^61^ Microarray data were normalized, background corrected, and log2 converted using the R package “affy” (https://bioconductor.org/packages/release/bioc/html/affy.html).^63^

The single-cell RNA-seq (scRNA-seq) data were analyzed using the “Seurat V4” R package (https://satijalab.org/seurat/).^62^ Briefly, cells with gene expression >500 genes and mitochondrial gene expression >25% were excluded. The ScaleData function based on variance stabilization (vst) is utilized to normalize raw counts. The principal component analysis (PCA) was utilized to reduce the dimensionality. We further used the R “Harmony” package (https://portals.broadinstitute.org/harmony/)^64^ to remove batch effects across different samples. Applying uniform manifold approximation and projection (UMAP) in Seurat, we identified discrete cell clusters and annotated them based on known cell type marker genes.

#### Identification of CAF-senescence-related genes correlated with PDAC prognosis

The cellular senescence scores of each cell in the scRNA-seq data were calculated by the UCell algorithm^20^ using the R package “irGSEA” (https://github.com/chuiqin/irGSEA).^65^ The gene set of cellular senescence was obtained from a previous study.^19^ Differential gene expression analysis in CAFs was performed using the “FindAllMarker” function in Seurat. CAF-upregulated genes were defined as those expressed in CAFs with a log2 fold change (logFC) higher than 0.5 compared to all other cell types. The correlation coefficient between each gene’s expression and the senescence scores in CAFs was calculated using the Spearman method. Then, the genes positively correlated with senescence scores (Spearman ρ > 0.2 and FDR< 0.05) were intersected with CAF-upregulated genes, which generated the CAF-senescence-related genes.

To evaluate the impact of senescence in CAFs on PDAC progression, SCAF abundance, and overall CAF abundance were assessed by the single-sample gene set enrichment analysis (ssGSEA) using the R package “GSVA” (https://github.com/rcastelo/GSVA).^66^ The CAF-upregulated genes (Table S2) were used for assessing overall CAF abundance, and the CAF-senescence-related gene signature (Table S3) was used for assessing SCAF abundance. The SCAF index was calculated by the ratio of SCAF enrichment scores to overall CAF enrichment scores.

Samples without survival data or overall survival (OS) of less than 30 days were removed. Firstly, the prognosis-associated genes in CAF-senescence-related genes were screened by univariate Cox regression. Then, three machine-learning algorithms, including least absolute shrinkage and selection operator (LASSO), RandomForest, and XGBoost, were implied to further screen genes tightly linked to PDAC prognosis using the R package “glmnet” (https://cran.r-project.org/web/packages/glmnet/index.html),^68^ “randomForest” (https://cran.r-project.org/web/packages/randomForest/index.html), and “caret” (https://cran.r-project.org/web/packages/caret/index.html).^67^

#### Establishment and validation of the CAF senescence score

To establish a prognostic index to predict the OS probabilities, the CAF senescence score (CSscore) of each patient was calculated by the formula:

CSscore=ΣiCoefficient(mRNAi)×Expression(mRNAi)

Then, PDAC patients in each dataset were divided into the high- and low-CSscore subsets by the median values. The Kaplan-Meier method was applied to carry out survival analysis using the R packages “survival” (https://cran.r-project.org/web/packages/survival/) and “survminer” (https://cran.r-project.org/web/packages/survminer/).

#### Functional enrichment analysis and cell-cell communication analysis

The CAF senescence signature was functionally annotated by calculating the enrichment of Gene Ontology (GO) terms (biological process, cellular component terms, molecular functions) and Reactome pathways using the R package “clusterProfiler” (https://github.com/YuLab-SMU/clusterProfiler).^69^ The enrichment scores of GO terms and Kyoto Encyclopedia of Genes and Genomes (KEGG) terms were assessed with the R package “GSVA” (https://github.com/rcastelo/GSVA). The Gene Set Enrichment Analysis (GSEA) was performed using the R packages “clusterProfiler” and “GseaVis” (https://github.com/junjunlab/GseaVis). The gene sets for Gene Set Variation Analysis (GSVA) and GSEA were downloaded from the Molecular Signatures Database (MSigDB) (https://www.gsea-msigdb.org/gsea/msigdb/)^77^ and the Enrichr database (https://maayanlab.cloud/Enrichr/).^78^^,^^79^^,^^80^ Differentially expressed gene (DEG) analysis between the high- and low-CSscore group was conducted using the “DESeq2” R package (https://bioconductor.org/packages/release/bioc/html/DESeq2.html).^70^

CAFs in the scRNA-seq dataset were divided into high- and low-senescent CAFs (H-SCAFs and L-SCAFs, respectively) based on the enrichment scores of the senescence signature. Cellchat (Version 1.6.1, https://github.com/sqjin/CellChat)^71^ was employed to analyze intercellular communication using the normalized gene expression matrix.

#### Immune infiltration analysis

To investigate the relationship between the CSscore and immune cell infiltration in PDAC samples, several well-established algorithms, including xCELL, ESTIMATE, quanTIseq, and ssGSEA were applied to assess the abundance of various immune cell populations within the TME using the R package “IOBR” (Version 0.99.9, https://github.com/IOBR/IOBR/).^72^ The differences in immune infiltration level assessed by those methods between high- and low-CSscore groups were analyzed with the Wilcoxon signed-rank test. The findings were visually represented in a complex heatmap using the R package “ComplexHeatmap” (https://github.com/jokergoo/ComplexHeatmap).^81^ The TIP (Tracking Tumor Immunophenotype) tool was applied to evaluate the abundance of tumor-infiltrating immune cells and assess the activity of the seven-step Cancer-Immunity Cycle.^35^

#### Therapy sensitivity analysis

To explore the predictive value of the CSscore signature for immunotherapy, we employed various computational tools and gene signatures. The R package “easier” (https://github.com/olapuentesantana/easier)^73^ was utilized to calculate scores for Cytotoxic activity (CYT),^36^ Davoli immune signature (Davoli_IS),^37^ and tertiary lymphoid structures (TLS)^38^ signature. Additionally, we utilized the Tumor Immune Dysfunction and Exclusion (TIDE) web tool^82^ to compute gene signature scores for several immune features, including IFN-γ (IFNG), Merck18 (T-cell-inflamed signature), CD8, MDSC, and CD274 signatures. The half-maximal inhibitory concentration (IC50) values for anti-tumor agents were calculated with the R package “oncoPredict” (https://github.com/maese005/oncoPredict).^74^

#### Preparation of conditioned medium (CM)

CAFs were seeded on 6-well plates at a density of 1× 10^5^ cells per well. After 24 h of attachment, cells were washed twice with PBS, and the medium was replaced with 2 mL of serum-free DMEM per well. For transfected CAFs, the culture medium was replaced with serum-free DMEM 48 h post-transfection. Following a 24-h incubation at 37°C, the CM was collected and filtered through 0.22 μm membrane syringe filters to remove cell debris. The filtered CM was stored at −80°C for future use.

#### Cell treatment

PANC-1 and BxPC-3 pancreatic cancer cell lines were cultured in fresh DMEM supplemented with 10% FBS one day before CM treatment. For experiments, cells were treated with a 1:1 mixture (v/v) of fresh media and CAF-CM for 48 h. Subsequently, colony formation, CCK-8, Transwell, and western blot assay were performed.

#### Plasmids Construction, transfection, and lentivirus infection

Viral particles expressing Notch1-shRNA and Notch1 were purchased from Genechem Corporation (Shanghai, China) and used to transduce PANC-1 and BxPC-3 according to the manufacturer’s protocol. After 48 h of infection, transduced cells were selected using puromycin for 7 days.

For silencing, the synthesized PLAU-specific shRNA sequences were inserted into the pLKO.1 vector. The sequences for RNAi are listed in Table S4. For expression, the PLAU sequences were PCR-amplified from cDNA and cloned into the pHAGE-CMV vector using primers listed in Table S5. These vectors, pMD2.G envelope, and psPAX packaging plasmids were co-transfected into 293-T cells with Lipofectamine 3000. After 72h, supernatants were collected, filtered (0.45μm), and used to transduce CAFs in the presence of polybrene (Sigma-Aldrich) for 8 h. Puromycin was used to select transduced cells for 7 days. Knockdown or overexpression efficiency was determined by Western blot and ELISA.

#### RNA isolation and qRT-PCR

Total RNA was extracted from cell lines with Trizol reagent (Invitrogen). cDNA was synthesized from the extracted RNA with HiScript III RT SuperMix (Vazyme). The qRT-PCR was performed using ChamQ Universal SYBR qPCR Master Mix (Vazyme). ACTB and Actb served as the internal reference genes. The used primers are listed in Table S6.

#### Western blot

RIPA lysis buffer supplemented with 1% protease inhibitor cocktail and 1% phenylmethylsulfonyl fluoride (PMSF) was used to extract the total cellular protein. Proteins were quantified via a BCA Protein Assay Kit (Thermo Fisher Scientific). Protein samples were mixed with 5× SDS-PAGE loading buffer (Servicebio) and heated at 95°C for 10 min. The boiled protein samples were then stored at −80°C for future use. Protein expression was assessed via western blot analysis, conducted according to the protocol described previously.^83^ Briefly, equal amounts of protein were separated on 10% SDS-polyacrylamide gels and transferred to methanol-activated PVDF membranes (0.45 μm, Millipore) using a wet transfer system. The membranes were blocked with 5% nonfat milk in TBST for 1 h at room temperature and then incubated with primary antibodies overnight at 4°C. Afterward, the membranes were washed three times with TBST (10 min each) and incubated with appropriate HRP-conjugated secondary antibodies for 1 h at room temperature. Protein blots were visualized using an enhanced chemiluminescence (ECL) detection kit (Abbkine). Monoclonal mouse anti-α-tubulin was used as an internal control. Primary antibodies included anti-p21 antibody (Santa cruz, sc-6246, 1:1000), antii-p53 antibody (Proteintech, 10442-1-AP, 1:5000), anti-α-SMA antibody (BOSTER, BM002, 1:2000), anti-FAP antibody (ABclonal, A23789, 1:1000), anti-Vimentin antibody (ABGENT, AX10005, 1:2000), anti-PLAU antibody (ABclonal, A2181, 1:1000), anti-Notch1 antibody (Proteintech, 20687-1-ap, 1:600), anti-Cleaveted Notch1 antibody (CST, 4147, 1:1000), anti-Hes1 antibody (ABclonal, A11718, 1:1000), anti-Hey1 antibody (Proteintech, 19929-1-AP, 1:2000), and anti-α-Tubulin antibody (Proteintech, 66031-1-Ig, 1:20000).

#### Enzyme-linked immunosorbent assay (ELISA)

The CM of PLAU-depleted SCAFs and PLAU-overexpressing CAFs were collected to measure the secreted protein levels of PLAU using the PLAU ELISA kit. The Human PLAU ELISA Kit and Mouse PLAU ELISA kit were purchased from BOSTER (Wuhan, China) and Hengyuan Biological Technology (Shanghai, China). Each sample was measured in duplicate cells. Concentrations were calculated according to the manufacturer’s instructions.

#### Cell migration and invasion assay

Transwell assay was conducted to evaluate the migratory and invasive capabilities of pancreatic cancer cells, employing 6.5 mm polycarbonate Transwell filters with 8 μm pores (Millipore, Billerica, MA, USA). In the migration assay, the upper chamber was seeded with 5× 10^4^ cells in serum-free DMEM, while the lower chamber contained DMEM supplemented with 10% FBS. Following a 36–48 h incubation period, the cells were fixed and quantified using microscopy. The invasion assay followed a similar protocol, with the additional step of uniformly coating the Transwell chamber with a layer of Matrigel before cell seeding. The experiments were performed in triplicate to ensure reproducibility.

#### Colony-formation assay

Cells were seeded in 6-well plates (500 cells/well) and incubated for 10 days. After being fixed with paraformaldehyde, colonies were stained with crystal violet solution for 30 min and quantified.

#### Cell proliferation assay

The cell proliferation assay was performed using the Cell Counting Kit-8 (YEASEN). Treated PANC-1 and BxPC-3 cells were seeded in 96-well plates at a density of 1.5× 10^3^ cells/well in 100 μL of culture medium. After incubation for the indicated periods (24, 48, 72, and 96 h), 10 μL of CCK-8 solution was added to each well. The plates were then incubated at 37°C in a 5% CO_2_ atmosphere for 2 h. The absorbance of each well was measured at 450 nm using a microplate reader.

#### Cellular senescence induction

A modified protocol was conducted according to the protocol described previously.^84^^,^^85^ Briefly, at day 0, seed CAFs at a density of 5× 10^5^ cells per 10 cm culture dish and allow them to attach for 24 h. At day 1, remove the supernatant and replace it with a growth medium containing 400 μM hydrogen peroxide (H_2_O_2_). After 2 h of treatment, wash the cells with PBS 3 times to remove residual H_2_O_2_, and then add fresh growth medium. Repeat the treatment at day 4 and day 7. Observe the cell morphological changes daily. When cell density exceeds 90%, subculture the cells at a 1: 2 ratio. The senescence was identified by qRT-PCR, SA-β-galactosidase staining, and western blot.

#### SA-β-galactosidase (SA-β-Gal) staining

Staining was carried out using the SA-β-Gal Staining Kit (Beyotime) according to the manufacturer’s protocol. Following the indicated assays in 12-well plates, cells were washed twice with PBS and fixed with 0.5 mL of β-Gal staining fixative solution for 15 min at room temperature. Subsequently, cells were washed three times with PBS for 3 min each, and incubated with 0.5 mL β-Gal staining buffer overnight at 37°C. Stained cells were visualized using light microscopy, and images were captured for quantitative analysis.

#### Immunofluorescence

Cells were seeded on glass slides in 24-well plates at a density of 2× 10^4^ cells/well and allowed to attach. The cells were washed twice with PBS and fixed with 4% paraformaldehyde for 20 min. Afterward, cells were washed three times with PBS and permeabilized with 0.1% Triton X-100 for 20 min at room temperature. To block non-specific sites, the cells were blocked with 4% FBS in PBS for 1 h and then incubated with primary antibodies diluted in 1% FBS in PBS overnight at 4°C. The following day, the cells were washed with PBS three times and incubated with secondary antibodies for 2 h in the dark followed by 30 min of DAPI incubation. Images were photographed with an LSM880 confocal microscope (Zeiss). Primary antibodies included anti-α-SMA antibody (BOSTER, BM002, 1:200), anti-FAP antibody (ABclonal, A23789, 1:200), and anti-Notch1 antibody (Proteintech, 20687-1-ap, 1:200).

#### Immunohistochemical analysis

Tumor tissues were fixed in 4% Paraformaldehyde for 24 h, dehydrated, and embedded in paraffin. The 4-μm sections were cut, baked at 60°C for 2 h, and then deparaffinized in xylene for 20 min. The sections were hydrated through an alcohol gradient and subjected to antigen retrieval in Tris-EDTA buffer (Proteintech) at 95°C for 20 min. When cooled to room temperature, slides were blocked with 3% hydrogen peroxide for 15 min, washed with PBS three times, and blocked with 10% goat serum for 10 min. Sections were then incubated with primary antibodies diluted in goat serum at 4°C for 12 h. After washing three times with PBS, sections were incubated with HRP-conjugated secondary antibodies (Proteintech) for 1 h and rewashed. Sections were stained using a diaminobenzidine (DAB) kit (Proteintech) for 5 min and rinsed in tap water. Representative images were captured using light microscopy at 400× magnification. The average number of positive cells was quantified from 5 random fields per section. Primary antibodies included anti-Ki67 antibody (CST, 9129, 1:400), anti-CD8 antibody (Abcam, ab209775, 1:2000), anti-CD206 antibody (CST, 24595, 1:500), and anti-Gr-1 antibody (Invitrogen, 14-5931-82, 1:200).

#### Flow cytometry

Single-cell suspensions were prepared from tumor tissues using the Tumor Dissociation Kit, mouse (Miltenyi Biotec) according to the manufacturer’s protocol. Lytic histiocyte cells (2× 10^6^ cells) were collected by centrifugation and resuspended in FACS buffer (PBS containing 0.5% fetal bovine serum). The cells were then incubated with Fc receptor blocking antibody (anti-CD16/CD32 antibody) for 15 min on ice. Subsequently, cells were stained according to the panels of interested populations. Stained cells were analyzed by the FACS Calibur machine with CellQuest software (BD Biosciences). The generated data were processed using FlowJo Software (Tree Star). The gating strategy for immune cells is shown in Figure S12. Antibodies were listed in the key resources table.

### Quantification and statistical analysis

Data are presented as mean ± standard deviation (SD). The Kaplan-Meier survival analysis was conducted with a Log rank test. Prognostic factors were identified through univariate and multivariate Cox proportional hazards regression analyses. Correlation coefficients were calculated using Spearman correlation analyses. Unpaired Student’s two-tailed *t*-test and Mann-Whitney test were utilized to evaluate the differences between the two groups. One-way analysis of variance (ANOVA) and the Kruskal-Wallis test were used for comparisons among three or more groups. two-way ANOVA was used to compare differences between groups on two independent variables. Two-sided Pearson’s chi-squared tests or Fisher’s exact tests were performed to assess differences in categorical variables between groups. Statistical analysis was performed using R software (Version 4.2.2) and GraphPad Prism (Version 10.2) software. *p* < 0.05 was set for statistical significance. ∗*p* < 0.05, ∗∗*p* < 0.01, ∗∗∗*p* < 0.001.
