# Supplementary material for: Revealing the role of cancer-associated fibroblast senescence in prognosis and immune landscape in pancreatic cancer
Source: iScience. 2024 Dec 16;28(1):111612. doi: 10.1016/j.isci.2024.111612 (PMC11742819; doi:10.1016/j.isci.2024.111612)
Supplement: Document S1. Figures S1–S12 and Tables S1–S7 [file mmc1.pdf]

iScience, Volume *28*

Supplemental information

Revealing the role of cancer-associated fibroblast senescence in prognosis and immune landscape in pancreatic cancer

Luyao Liu, Hai Huang, Bin Cheng, Huaping Xie, Wang Peng, Haochen Cui, Jingwen Liang, Mengdie Cao, Yilei Yang, Wei Chen, Ronghua Wang, and Yuchong Zhao

Supplementary Figures and Legends:

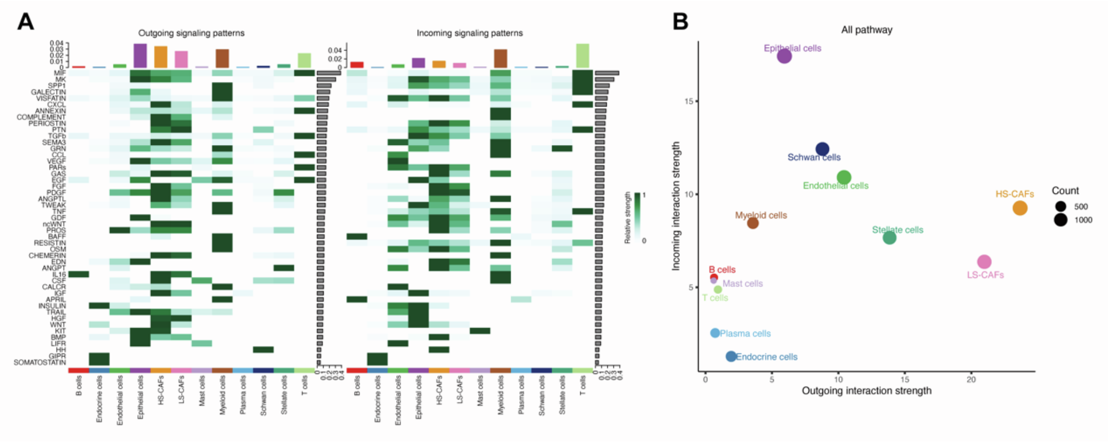

Figure. S1. Analysis of signaling pathways involved in cell-cell interactions, related to Figure 1. A-B. Heatmap and dot plot showing the outgoing (**A**) and incoming (**B**) signal strength of each signaling pathway among different cell types in PDAC.

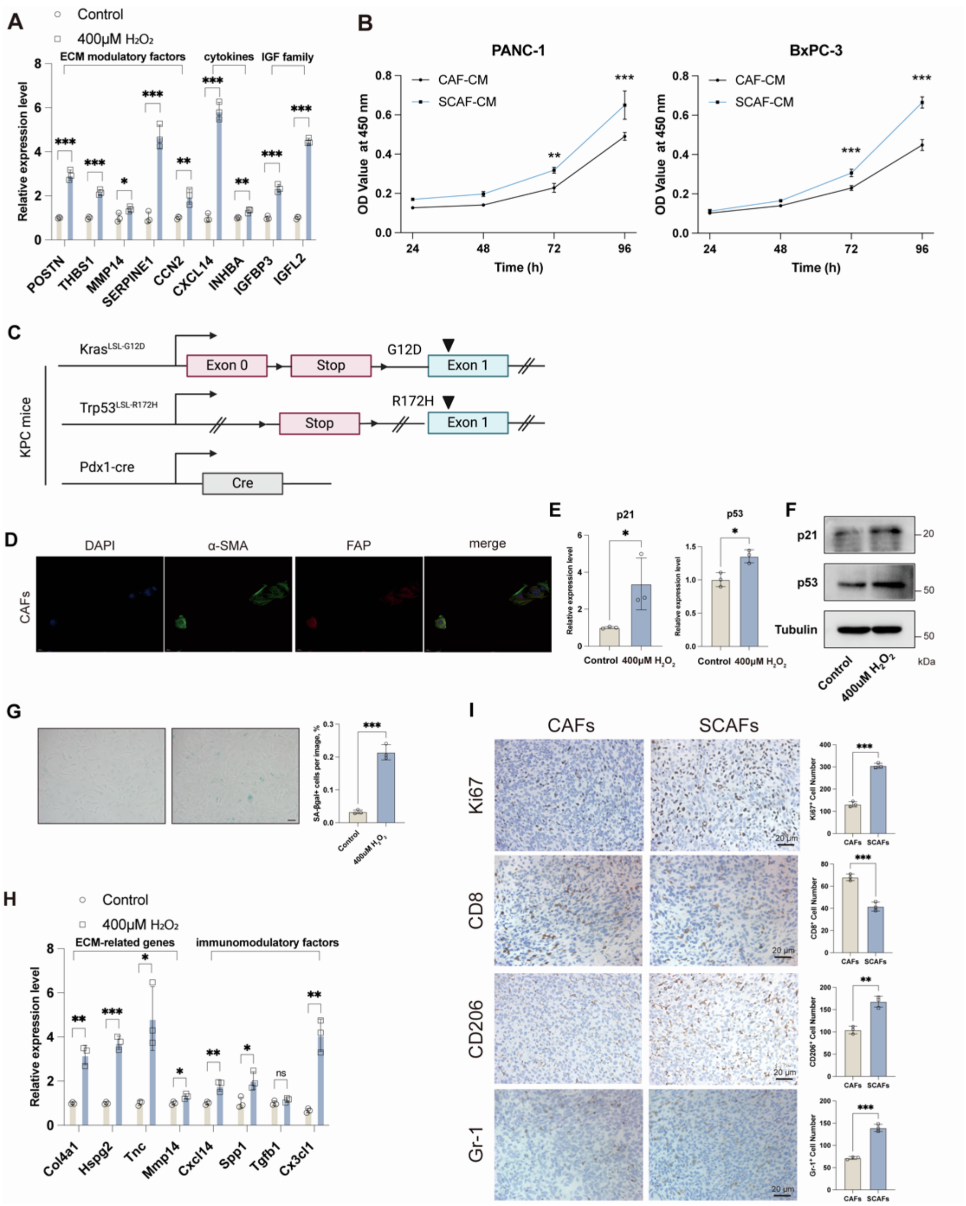

Figure. S2. SCAFs promote PDAC progression and impair anti-tumor immunity, related to Figure 2. A. Expression levels of genes encoding ECM modulatory factors, cytokines, and IGF family members in human CAFs and H_2O_2-induced SCAFs were measured by qRT -PCR. **B.** The proliferation ability of PDAC cells was assessed using CCK8 assay. **C.** Scheme of the KPC mice model. **D.** Representative images of immunofluorescence for α-SMA and FAP in CAFs from KPC mice. Scale bar, 20 μm. **E.** Relative mRNA expression of p21 and p53 in CAFs from KPC mice treated with 400 μM H_2O_2. **F**. Protein expression of p21 and p53 in CAFs from KPC mice treated with 400 μM H_2O_2 was measured by western blot. **G**. SA-β-Gal staining of CAFs from KPC mice treated with 400 μM H_2O_2. Scale bar, 20 μm. **H.** Expression levels of ECM-related genes and genes encoding immunomodulatory factors in mouse CAFs and H_2O_2-induced SCAFs were measured by qRT-PCR. **I.** Representative immunohistochemical staining and statistical analysis of Ki67, CD8, CD206, and Gr-1 in different groups. Scale bar, 20 μm.

Data are represented as mean ± SD. *$p < 0.05$, **$p < 0.01$, ***$p < 0.001$.

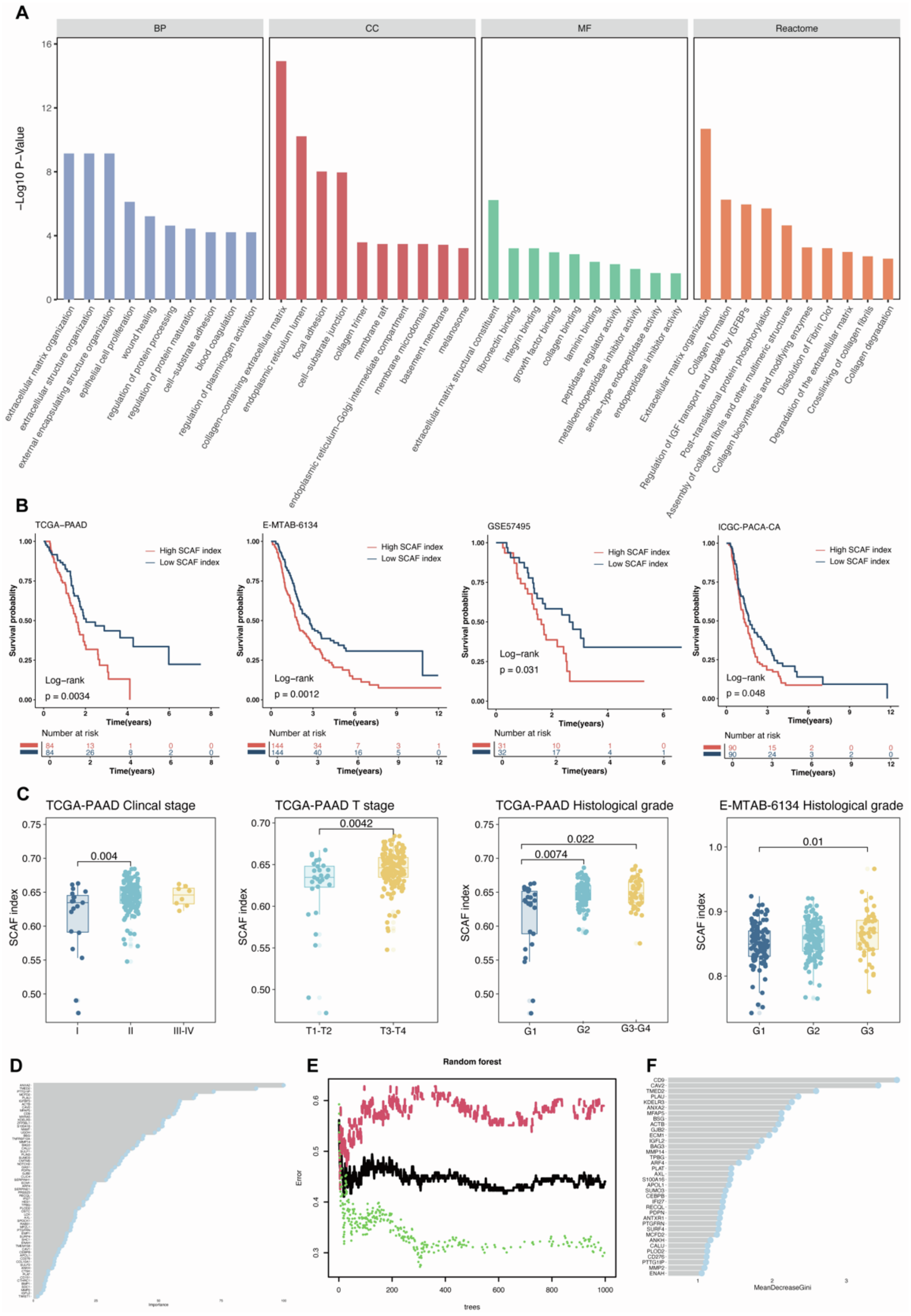

Figure. S3. A gene signature based on CAF senescence improves prognostic prediction in PDAC, related to Figure 3. A. Functional enrichment analysis of GO terms and Reactome pathways of the CAF senescence signature. **B**. Kaplan-Meier survival analysis of OS for patients with high and low SCAF index grouped by the median value in TCGA-PAAD, E-MTAB-6134, GSE57495, and ICGC-PACA-CA cohorts. **C.** Difference analysis of the distribution of the SCAF index in different clinical stages, T stages, and histological grades of PDAC patients. **D.** 68 genes were selected by XGBoost based on the importance of the feature. **E.** Random Forest error rate versus the number of classification trees. **F.** 36 genes were selected by RandomForest based on the importance of the feature.

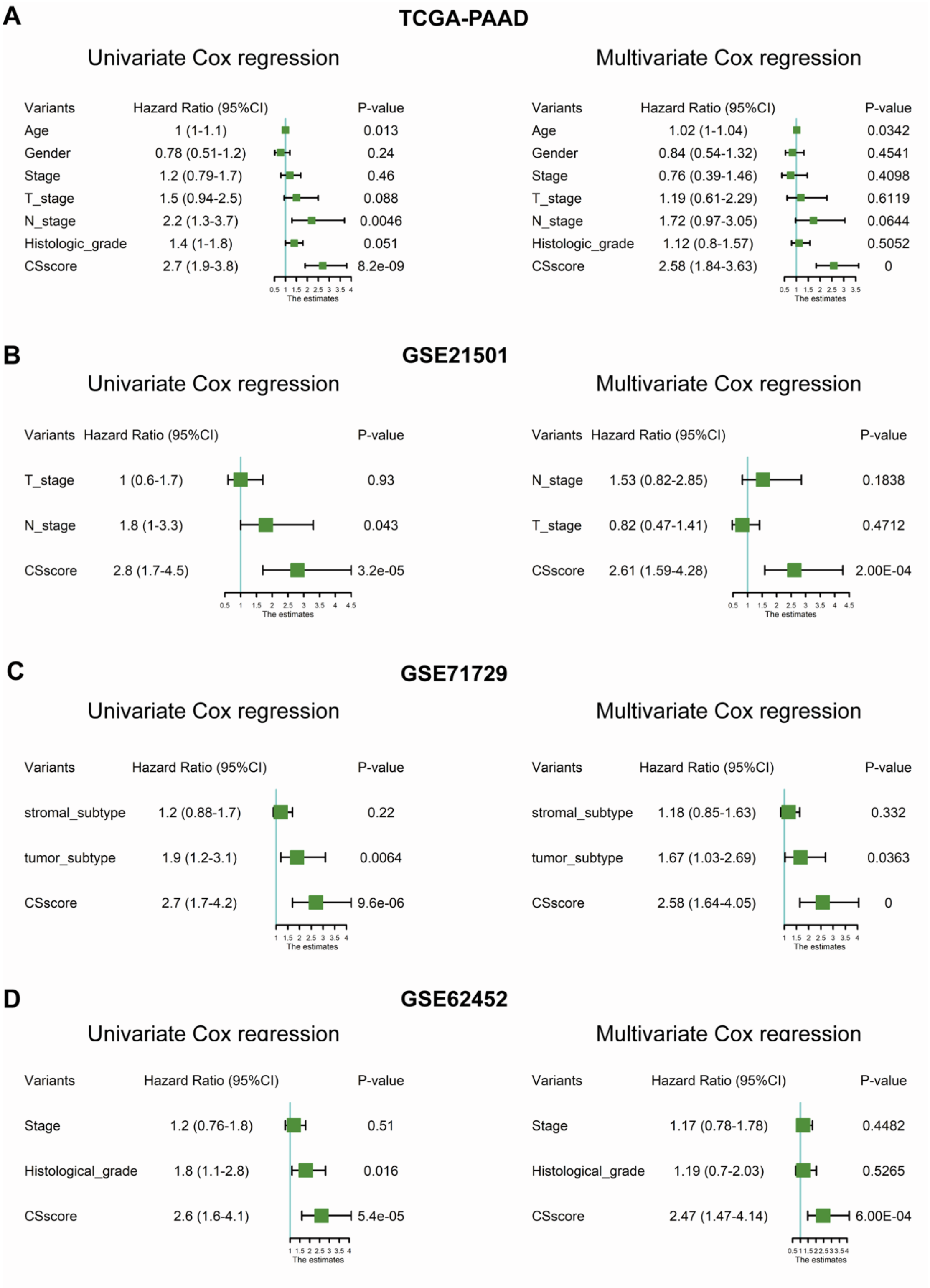

Figure. S4. Univariate and multivariate Cox regression analysis in four datasets, related to Figure 5. A-D. Forest plots of the results of the univariate and multivariate Cox regression analysis for TCGA-PAAD (**A**), GSE21501 (**B**), GSE71729 (**C**), and GSE62452 (**D**) cohorts, respectively.

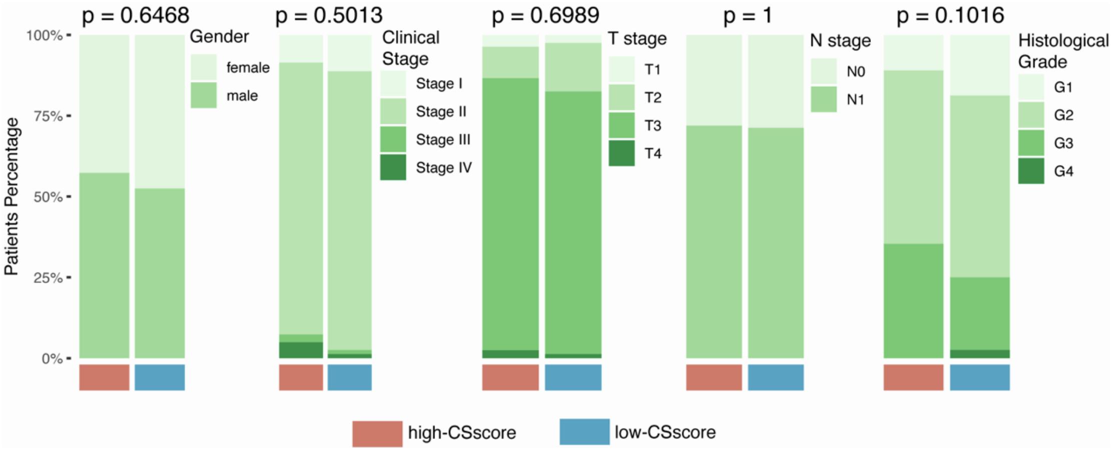

Figure. S5. Bar chart representing the compositions of different clinical characteristics by high- and low-CSscore group, related to Figure 7.

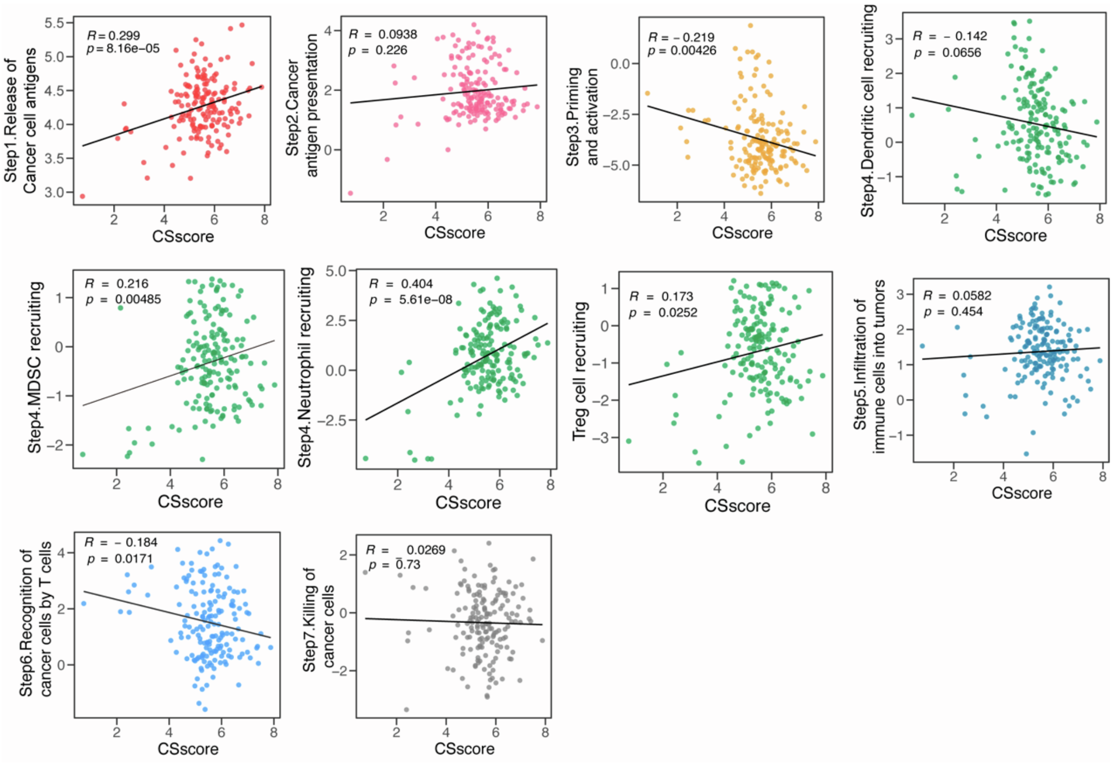

Figure. S6. The relationship between the CSscore and anti-cancer immune activities was assessed by the "TIP" tool in the TCGA-PAAD dataset, related to Figure 8.

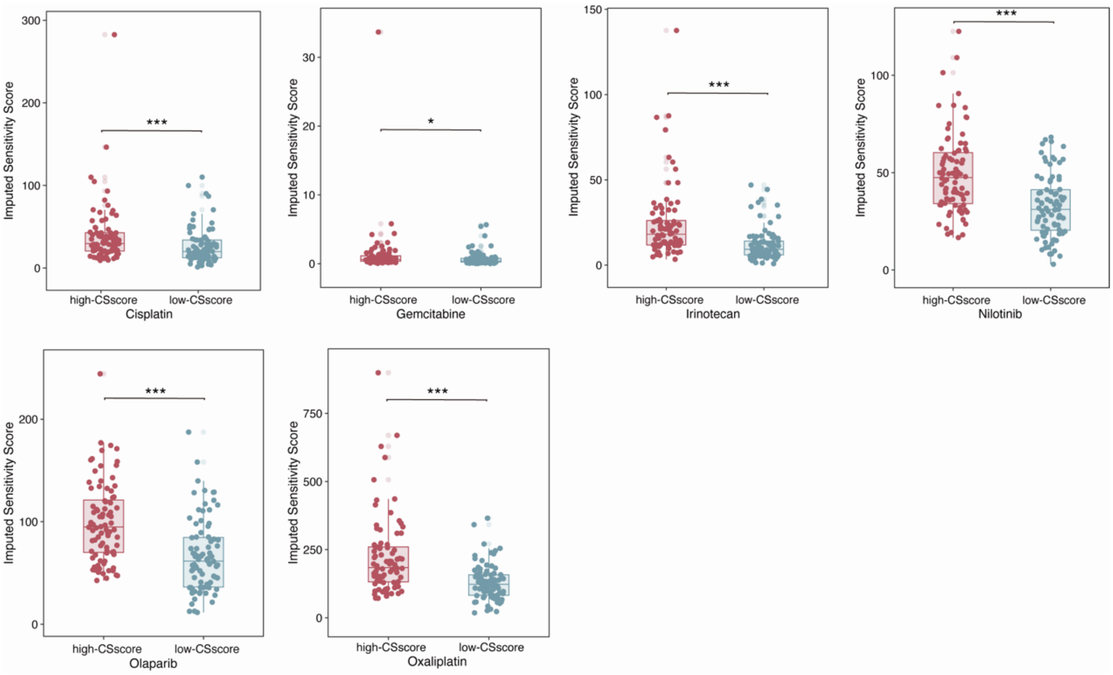

Figure. S7. The differences in the chemotherapeutic sensitivity based on IC50 for six drugs between the high- and low-CSscore groups, related to Figure 8.

*p < 0.05, **p < 0.01, ***p < 0.001.

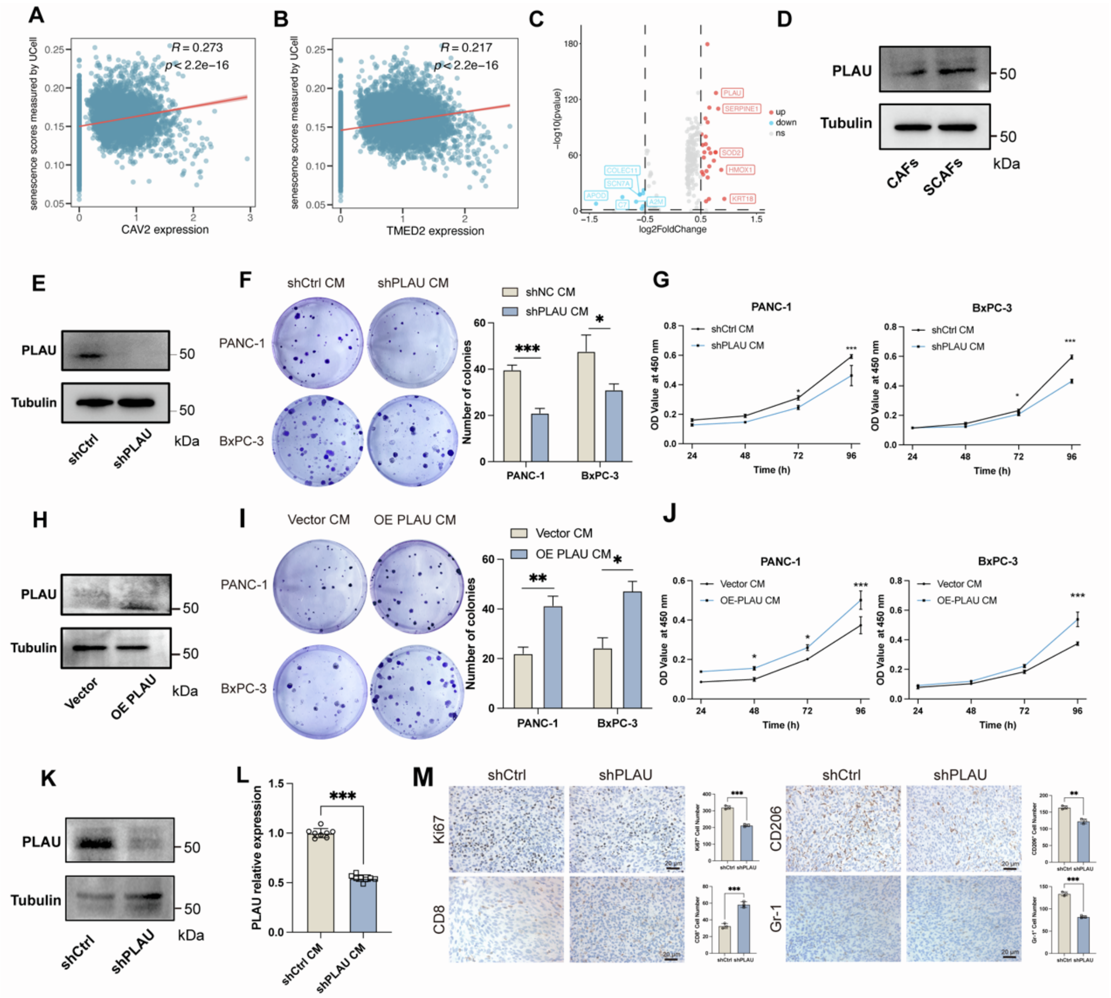

Figure. S8. SCAFs derived PLAU promotes PAAD progression, related to Figure 9. A. The relationship between CAV2 expression and UCell senescence scores in CAFs. **B.** The relationship between TMED2 expression and UCell senescence scores in CAFs. **C.** Volcano plot of DEGs in HS-CAFs and LS-CAFs. **D.** Protein expression for PLAU in indicated CAFs. **E.** PLAU knockdown in human SCAFs was verified by western blot. **F.** The proliferation ability of the indicated cells was measured by colony formation assay. **G.** The proliferation ability of the indicated cells was measured by CCK-8 assay. **H.** PLAU overexpression in human CAFs was verified by western blot. **I.** The proliferation ability of the indicated cells was measured by colony formation assay. **J.** The proliferation ability of the indicated cells was measured by CCK-8 assay. **K-L.** PLAU knockdown in mouse SCAFs was verified by western blot (**K**) and ELISA (**L**). **M.** Representative immunohistochemical staining and statistical analysis of Ki67, CD8, CD206, and Gr-1 in different groups. Scale bar, 20 μm.

Data are represented as mean ± SD. $*p < 0.05$, $**p < 0.01$, $***p < 0.001$.

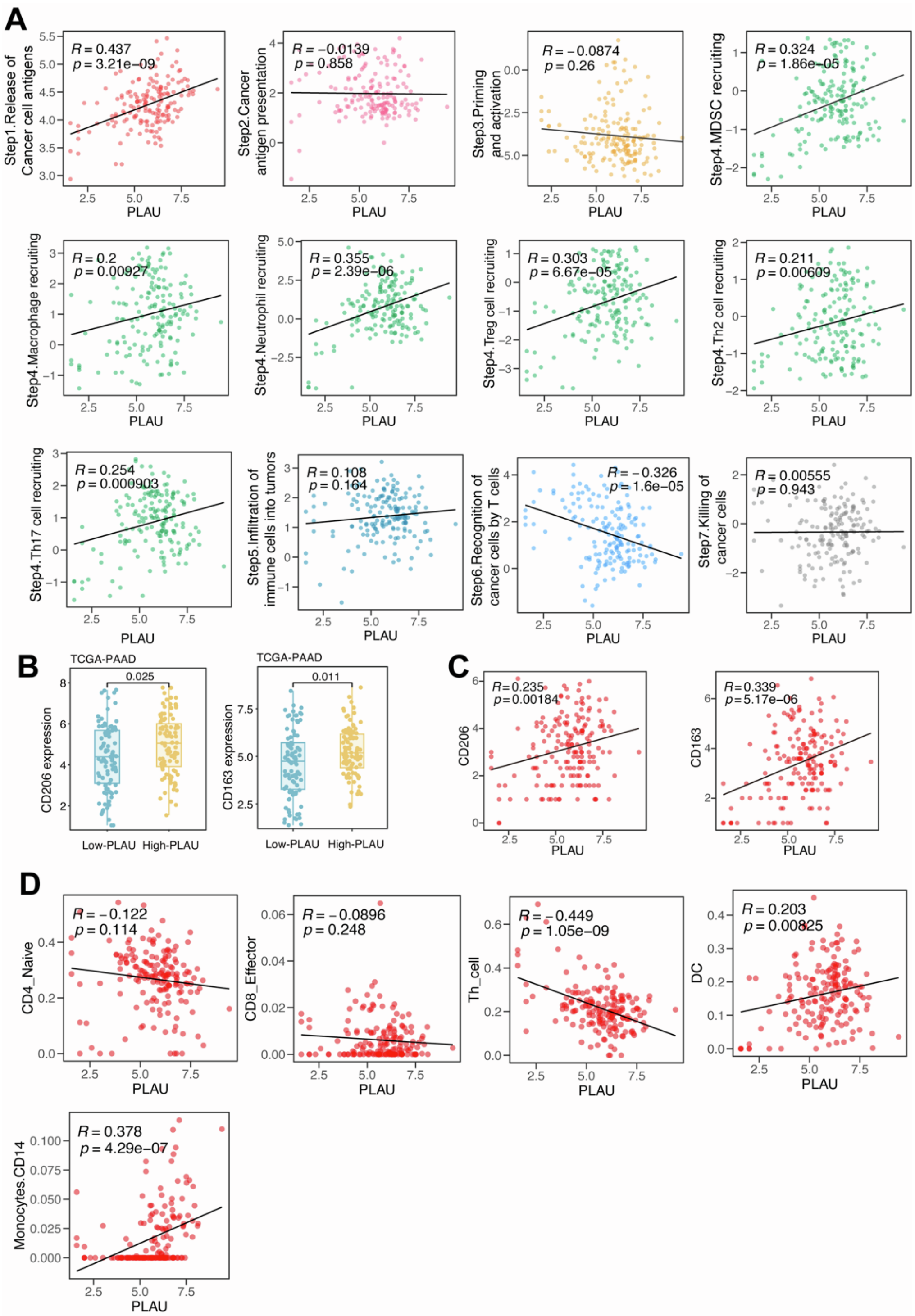

Figure. S9. Identification of PLAU as an immunosuppressive regulator in PAAD via bioinformatic analysis, related to Figure 9. A. The relationship between PLAU expression and anti-cancer immunity activities was assessed by the "TIP" tool in the TCGA-PAAD dataset. **B.** Differences in the expression levels of CD206 and CD163 between the High- and Low-PLAU expression groups (based on the median values). **C.** The relationship between PLAU expression and CD163, CD206 expression in TCGA-PAAD dataset. **D**. The relationship between PLAU expression and indicted immune cell abundance was assessed by the "TIP" tool in the TCGA-PAAD dataset.

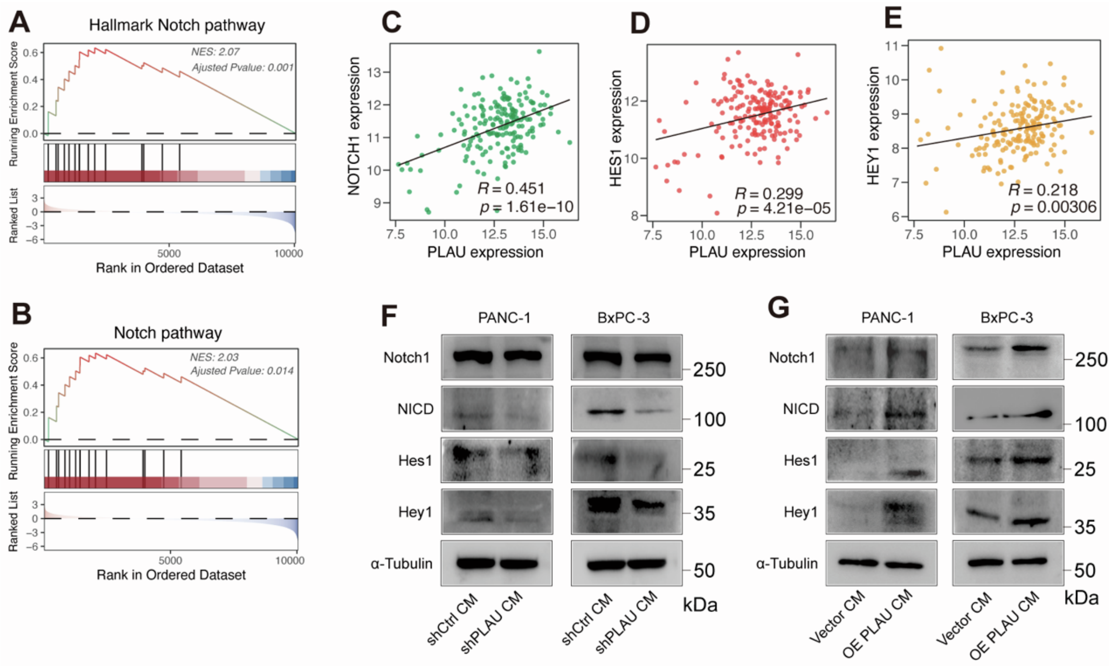

Figure. S10. SCAF-derived PLAU activates the Notch1 signaling pathway in PDAC cells, related to Figure 9. A-B. GSEA revealed enrichment of the Notch pathway from the Hallmark gene set (**A**) and the Enrichr database (**B**) in the group with high-PLAU expression in the TCGA-PAAD cohort. **C-E.** The relationship between PLAU expression and the expression of key molecules of the Notch pathway, including Notch1 (**C**), Hes1 (**D**), and Hey1 (**E**) in the TCGA-PAAD dataset. **F-G**. Protein expression of Notch1, NICD, Hes1, and Hey1 in different groups was measured by western blot.

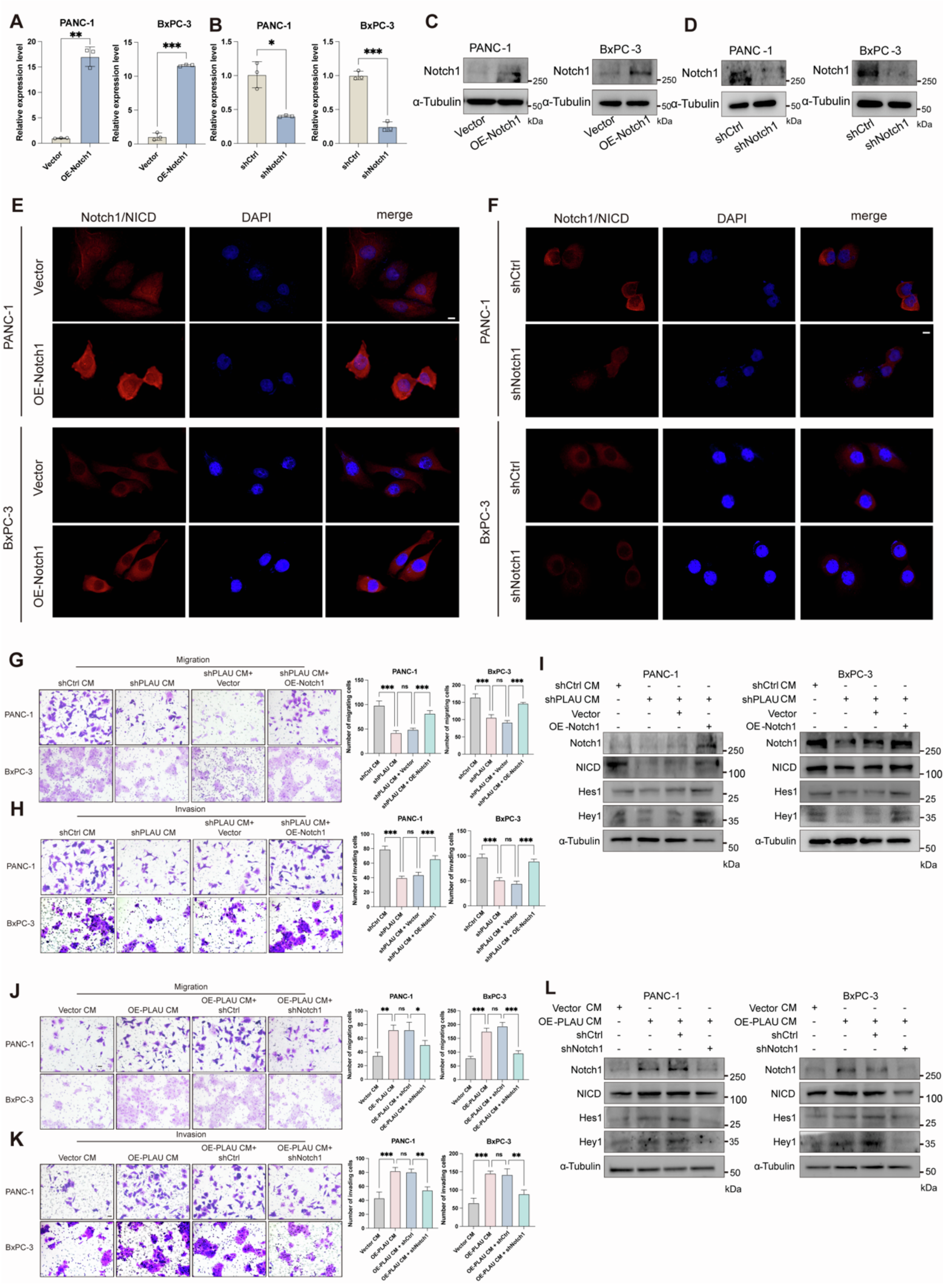

Figure. S11. SCAF-derived PLAU promotes pancreatic cancer cell migration by activating the Notch1 signaling pathway, related to Figure 9. A-B. Efficiency of Notch1 stable overexpression (**A**) and knockdown (**B**) was verified in PANC-1 and BxPC-3 cell lines by real-time PCR. **C-D**. Efficiency of Notch1 stable overexpression (**C**) and knockdown (**D**) was verified in PANC-1 and BxPC-3 cell lines by western blot. **E**. IF images of Notch1/NICD in Notch1-overexpressing and control cells. Scale bar, 100 μm. **F.** IF images of Notch1/NICD in Notch1-knockdown and control cells. Scale bar, 100 μm. **G-**

H. The migration (**G**) and invasion (**H**) abilities of the indicated PDAC cells were measured by Transwell assay. Scale bar, 20 μm. **I.** Protein expression of Notch1, NICD, Hes1, and Hey1 in different groups was measured by western blot. **J-K.** The migration (**J**) and invasion (**K**) abilities of the indicated PDAC cells were measured by Transwell assay. Scale bar, 20 μm. **L.** Protein expression of Notch1, NICD, Hes1, and Hey1 in different groups was measured by western blot.

Data are represented as mean ± SD. *p < 0.05, **p < 0.01, ***p < 0.001.

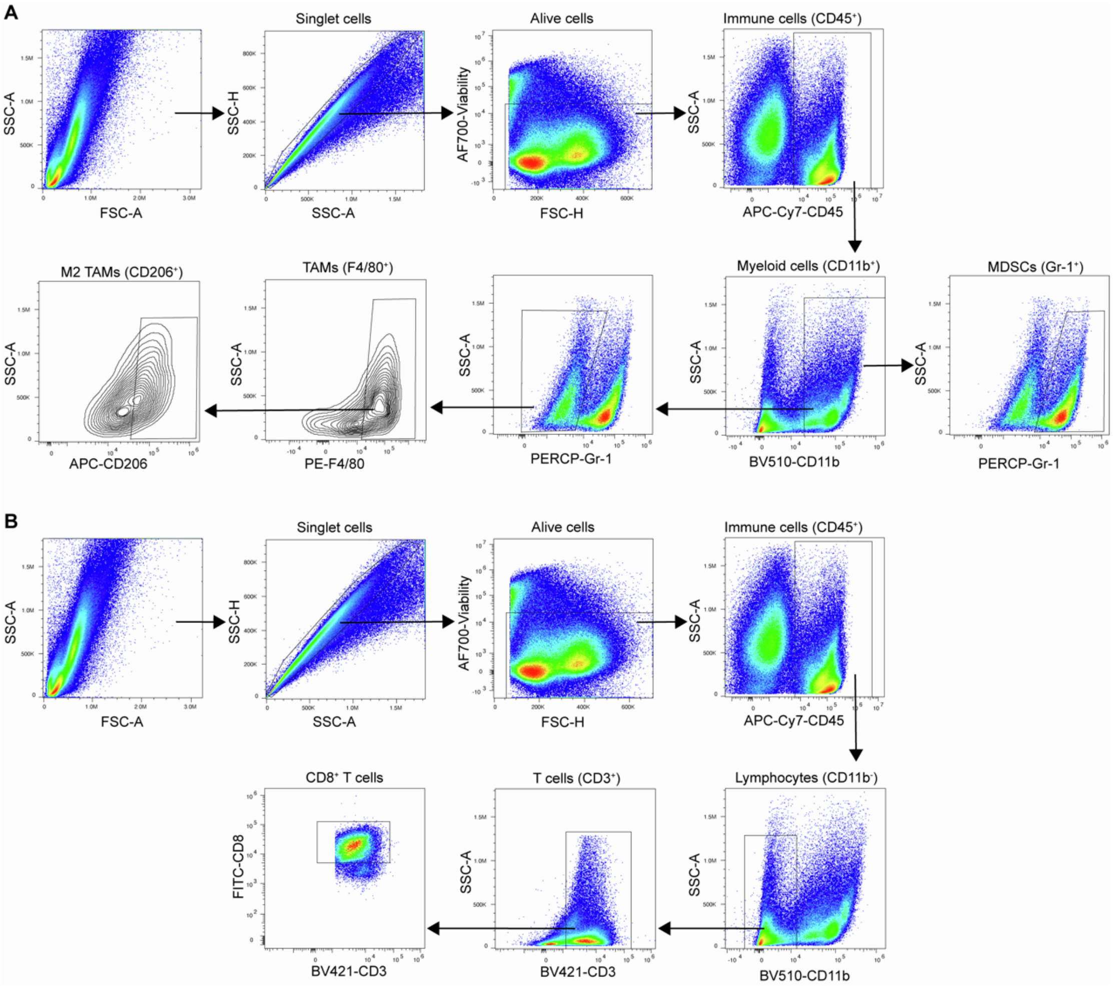

Figure. S12. Gating strategy for flow cytometry, related to Figure. 2L-O and Figure. 9K-N.

A. Gating strategy of $CD11b^+Gr1^+$ MDSCs and $F4/80^+CD206^+$ M2 macrophages in tumor tissues of C57BL/6 mice. **B**. Gating strategy of $CD45^+CD3^+$ T cells and $CD3^+CD8^+$ T cells in tumor tissues of C57BL/6 mice.

Supplementary Tables:

Table S1. Gene list of SenMayo, related to Figure 1.

ACVR1B	ANG	ANGPT1	ANGPTL4	AREG	AXL	BEX3
BMP2	BMP6	C3	CCL1	CCL13	CCL16	CCL2
CCL20	CCL24	CCL26	CCL3	CCL3L1	CCL4	CCL5
CCL7	CCL8	CD55	CD9	CSF1	CSF2	CSF2RB
CST4	CTNNB1	CTSB	CXCL1	CXCL10	CXCL12	CXCL16
CXCL2	CXCL3	CXCL8	CXCR2	DKK1	EDN1	EGF
EGFR	EREG	ESM1	ETS2	FAS	FGF1	FGF2
FGF7	GDF15	GEM	GMFG	HGF	HMGB1	ICAM1
ICAM3	IGF1	IGFBP1	IGFBP2	IGFBP3	IGFBP4	IGFBP5
IGFBP6	IGFBP7	IL10	IL13	IL15	IL18	IL1A
IL1B	IL2	IL32	IL6	IL6ST	IL7	INHA
IQGAP2	ITGA2	ITPKA	JUN	KITLG	LCP1	MIF
MMP1	MMP10	MMP12	MMP13	MMP14	MMP2	MMP3
MMP9	NAP1L4	NRG1	PAPPA	PECAM1	PGF	PIGF
PLAT	PLAU	PLAUR	PTBP1	PTGER2	PTGES	RPS6KA5
SCAMP4	SELPLG	SEMA3F	SERPINB4	SERPINE1	SERPINE2	SPP1
SPX	TIMP2	TNF	TNFRSF10C	TNFRSF11B	TNFRSF1A	TNFRSF1B
TUBGCP2	VEGFA	VEGFC	VGF	WNT16	WNT2	

Table S2. Gene list of CAF-upregulate genes, related to Figure 3.

AAMDC	ABCA1	A2M	ABI2	ACTA2	ACTB	ACTG1
ADAMTS12	ADAMTS2	ACTN1	ADD1	ADH5	ADI1	AEBP1
AHCYL1	AHNAK	AGTRAP	AIDA	AK1	AKAP12	AKR1A1
AKT3	ALDH1A1	AKR7A2	ALDH1A3	ALKBH7	ANAPC11	ANGPTL2
ANKRD10	ANO6	ANKH	ANTXR1	ANTXR2	ANXA2	ANXA5
AOPEP	AP2M1	ANXA6	AP2S1	AP3S1	APBB2	APOD
APP	ARF4	APOL1	ARHGAP1	ARHGAP21	ARID5B	ARL1
ARL2BP	ARL3	ARL2	ARMCX3	ASAP1	ASPH	ASPN
ATN1	ATOX1	ATL3	ATP5PF	ATP6AP2	ATP6V0E1	ATRAID
AZI2	BACE1	AXL	BAD	BAG3	BANF1	BASP1
BEX3	BGN	BDH2	BHLHE41	BICC1	BMP1	BMPR2
BOC	BORCS7	BNC2	BRI3	BSG	BST2	C11orf96
C1orf122	C1QTNF3	C12orf57	C1R	C1S	C3	C4orf3
CALD1	CALR	C5orf15	CALU	CAMK2D	CAMLG	CAPZB
CASC4	CAV1	CARHSP1	CAV2	CAVIN1	CAVIN3	CBX1
CCDC80	CCDC85B	CBX5	CCDC90B	CCN1	CCN2	CCPG1
CD276	CD302	CD151	CD63	CD81	CD9	CD99
CDH11	CDIPT	CDC42BPA	CDK14	CDK2AP1	CDK4	CDKN1C
CEBPD	CERCAM	CEBPB	CETN2	CFAP36	CFAP97	CFH

CHD9	CHID1	CFI	CHPF	CISD1	CKAP4	CLEC11A
CLMP	CLU	CLIC4	CMTM3	CNIH1	CNN2	CNN3
COL10A1	COL11A1	CNPY2	COL12A1	COL14A1	COL16A1	COL18A1
COL1A2	COL3A1	COL1A1	COL4A1	COL4A2	COL5A1	COL5A2
COL6A2	COL6A3	COL6A1	COL8A1	COL8A2	COLEC12	COMP
COPB2	COPS8	COMT	COPS9	COPZ2	COX7A1	COX7A2L
CPQ	CREB3L2	CPE	CRIM1	CRISPLD2	CRTAP	CSGALNACT2
CST3	CTDSP2	CSRP1	CTDSPL	CTHRC1	CTNNB1	CTSF
CTSO	CTTN	CTSK	CUEDC2	CUTA	CXCL12	CXCL14
CYB5R3	CYBRD1	CYB5A	CYP1B1	CYTH3	CZIB	DAB2
DAP	DCN	DAD1	DCTN2	DCTN3	DDAH2	DDR2
DIO2	DKK3	DESI2	DLC1	DMAC1	DNAJB4	DNAJC15
DPYSL2	DPYSL3	DOCK1	DSE	DST	DSTN	DTWD1
DYNC1I2	DYNC1LI2	DUT	DYNLL1	ECM1	ECM2	EDF1
EDNRA	EEA1	EDIL3	EEF2	EFEMP1	EFEMP2	EGR1
EID1	EIF4G3	EHD2	ELN	ELOB	EMC2	EMILIN1
EMP2	ENAH	EMP1	ENG	EPB41L2	ERCC1	ERLEC1
ETV1	EVA1B	ESD	FAM114A1	FAM162A	FAM200B	FAM3C
FARP1	FAT1	FAP	FBLN1	FBLN2	FBLN5	FBN1
FERMT2	FGF7	FBXO32	FGFR1	FHL2	FIBIN	FILIP1L
FKBP10	FKBP14	FIS1	FKBP7	FKBP9	FLNA	FMOD
FNBP1L	FNDC1	FN1	FNDC3B	FOS	FOSB	FOXO1
FRMD6	FRZB	FOXO3	FSTL1	FTX	FUNDC2	FZD1
GABARAPL2	GADD45A	GABARAP	GADD45B	GALNT1	GANAB	GAS1
GASK1B	GDI2	GAS6	GEM	GGT5	GINM1	GJA1
GLG1	GLI3	GJB2	GLIS2	GLT8D1	GLT8D2	GNB4
GNG12	GOLGA2	GNG11	GOLGA3	GOLIM4	GOLM1	GOPC
GPC1	GPX8	GPAA1	GREM1	GRHPR	GSN	GSTM3
GTF2H5	GXYLT2	GSTP1	H2AFJ	HACD3	HCFC1R1	HDLBP
HEG1	HES1	HEBP1	HMCN1	HNMT	HOOK3	HOPX
HSP90B1	HSPA1A	HSBP1	HSPA1B	HSPB1	HSPG2	HTRA1
IAH1	ID3	HTRA3	IER2	IFI27	IFI27L2	IFI6
IFT20	IGF1R	IFITM3	IGFBP3	IGFBP4	IGFBP5	IGFBP6
IGFL2	IKBIP	IGFBP7	IL1R1	IL6ST	ILK	IMPDH2
ISCU	ISG15	INHBA	ISLR	ISOC2	ITGA1	ITGA11
ITGB1	ITGB1BP1	ITGAV	ITGB5	ITGBL1	ITM2B	ITM2C
JAM3	JOSD2	ITPRIPL2	JUN	KANK2	KCNE4	KCNQ1OT1
KDELR2	KDELR3	KDELR1	KDM5B	KDSR	KIAA1217	KIDINS220
KLF10	KLF9	KIFAP3	LAMA2	LAMA4	LAMB1	LAMB2
LAPTM4A	LATS2	LAMC1	LEPROT	LGALS1	LGALS3BP	LHFPL6
LMAN1	LMCD1	LIMA1	LMO4	LOX	LOXL1	LPP
LSAMP	LTBP1	LRP1	LTBP2	LTBP3	LTBP4	LUM
MAGED1	MAGED2	LY6E	MAGI2-AS3	MAN1A1	MANF	MAP1A

MAP1LC3A	MAP4	MAP1B	MAP4K5	MARCKS	MARVELD1	MATN3
MBTPS1	MCFD2	MBNL2	MCRIP1	MDK	MED13L	MEG3
MFAP4	MFAP5	MFAP2	MFGE8	MGP	MGST3	MIB1
MICOS10	MICOS13	MICAL2	MICU1	MIF	MINDY2	MIR100HG
MIR99AHG	MITF	MIR4435-2HG	MMP11	MMP14	MMP2	MORF4L1
MOXD1	MPLKIP	MORF4L2	MPZL1	MRC2	MRFAP1	MRPL40
MRPS21	MSRB2	MRPL51	MSRB3	MT1E	MT2A1	MTCH1
MXRA5	MXRA7	MTR	MXRA8	MYDGF	MYH10	MYH9
MYL12B	MYL6	MYL12A	MYL6B	MYL9	MYLK	MYO1C
MYO1E	MYOF	MYO1D	NAA38	NAP1L1	NBDY	NBL1
NCOR2	NDN	NCKAP1	NDUFA11	NDUFA12	NDUFA13	NDUFA4
NDUFC2	NDUFS4	NDUFB7	NEDD8	NENF	NEXN	NF1
NFIA	NFIB	NFE2L1	NFIC	NFIX	NFYB	NID1
NME3	NME4	NME2	NNMT	NORAD	NOTCH2	NPTN
NR2F2	NR4A1	NR2F1	NREP	NRP2	NTAN1	NTM
NUCB1	NUCB2	NUAK1	NUCKS1	NUPR1	OAF	OLFML2B
OMD	OS9	OLFML3	OST4	OSTC	P3H1	P3H3
P4HB	PALLD	P4HA1	PAM	PARK7	PARVA	PAWR
PCDH7	PCMTD1	PBX1	PCOLCE	PDGFC	PDGFRA	PDGFRB
PDIA3	PDIA6	PDGFRL	PDLIM2	PDLIM3	PDLIM4	PDLIM5
PDPN	PEA15	PDLIM7	PEAK1	PEBP1	PGAM1	PGLS
PHB2	PHC2	PGRMC1	PHF14	PHLDA1	PHLDA3	PHLDB1
PKD2	PKIG	PHPT1	PLAC9	PLAT	PLAU	PLEC
PLOD1	PLOD2	PLIN3	PLPP3	PLS3	PLSCR4	PLTP
PLXNB2	PMP22	PLXDC2	PODN	PODNL1	POLR2F	POLR2L
PPFIBP1	PPIB	POSTN	PPIC	PPP1CC	PPP2CB	PRDX2
PRDX5	PRDX6	PRDX4	PRELP	PRKG1	PRRX1	PRSS23
PSMB1	PSMB5	PSD3	PTEN	PTGFRN	PTK7	PTMS
PTS	PTTG1IP	PTPN14	PWWP3A	PXDN	RAB13	RAB2A
RAB34	RABAC1	RAB31	RAI14	RAMP1	RARRES1	RARRES2
RASSF8	RBFOX2	RASAL2	RBMS3	RBP1	RBPMS	RCN1
RCN3	RECQL	RCN2	REEP3	REXO2	RHOBTB3	RHOC
RNASE4	RNASEH2C	RIN2	RND3	RNH1	ROCK2	RPS27L
RRBP1	RTL8C	RRAS	RTN4	RUFY3	RUNX1	RUNX1T1
RWDD1	S100A13	RUNX2	S100A16	SCARB2	SCPEP1	SDC1
SDF4	SEC11A	SDC2	SEC13	SEC23A	SEC24D	SEC31A
SEC61B	SEC61G	SEC61A1	SEC62	SEC63	SELENOF	SELENOM
SELENOS	SELENOW	SELENOP	SEMA3C	SEPTIN10	SEPTIN11	SEPTIN2
SEPTIN8	SERF2	SEPTIN7	SERPINE1	SERPINF1	SERPING1	SERPINH1
SFRP4	SGCB	SFRP2	SGCE	SH3BGRL	SH3D19	SH3PXD2A
SHC1	SLC38A10	SH3PXD2B	SLC38A2	SLC39A1	SLC39A6	SLC39A7
SLC6A6	SMAD5	SLC44A1	SMARCA1	SMIM3	SMOC2	SNHG29

SNX3	SNX9	SNHG32	SORBS3	SOX4	SPARC	SPARCL1
SPATS2L	SPIN1	SPART	SPOCK1	SPON1	SPON2	SPRY1
SRPX2	SSPN	SRM	SSR2	SSR3	ST5	STAT2
SULF1	SULF2	STMP1	SUMO2	SUMO3	SURF4	SVIL
SYNE1	TAGLN	SWI5	TAX1BP3	TCEAL3	TCEAL4	TCEAL8
TCF12	TCF4	TCEAL9	TEAD1	TGFB1I1	THBS1	THBS2
TIMM13	TIMM8B	THY1	TIMP1	TIMP2	TIMP3	TJP1
TMED2	TMED3	TMED10	TMED9	TMEM109	TMEM119	TMEM14C
TMEM167A	TMEM176A	TMEM158	TMEM204	TMEM230	TMEM248	TMEM258
TMEM30A	TMEM35B	TMEM263	TMEM43	TMEM45A	TMEM47	TMEM59
TMEM98	TMSB10	TMEM87A	TNFRSF12A	TNFRSF1A	TNS1	TNS3
TPBG	TPM1	TP53I3	TPM2	TPM4	TRIO	TRIP11
TSHZ2	TSPAN4	TSC22D1	TSPAN9	TTC3	TTC37	TUBA1A
TUBB	TUBB6	TUBA1B	TWIST1	TWSG1	TXNDC15	TXNDC5
UBE2E2	UBXN4	UACA	UGDH	UNC5B	UQCRQ	USO1
VAMP5	VAT1	USP22	VCAN	VCL	VEGFB	VGLL4
VKORC1	VMP1	VIM	WASF2	WASL	WBP1L	WDR13
WDR83OS	WLS	WDR45	WWTR1	YAP1	YBX3	YIF1A
ZBTB20	ZC2HC1A	ZBTB16	ZCCHC24	ZCRB1	ZEB1	ZFHX3
ZFP36L1	ZMAT3	ZFHX4	ZNF106	ZNF428	ZYX	

Table S3. Gene list of CAF senescence signature, related to Figure 3.

ACTB	ACTG1	AKR7A2	ALDH1A3	ANGPTL2	ANKH	ANTXR1
ANXA2	APOL1	ARF4	ARL2BP	AXL	BAG3	BASP1
BMP1	BSG	C11orf96	C1QTNF3	C3	C5orf15	CALU
CAV1	CAV2	CD151	CD276	CD63	CD9	CDK2AP1
CEBPB	CHPF	CKAP4	CLIC4	CLMP	CMTM3	CNN3
COL10A1	COL6A1	COL8A2	COLEC12	COPS8	CTDSP2	CTHRC1
CTSK	CXCL12	DNAJB4	DPYSL3	ECM1	EFEMP1	EGR1
EMP1	ENAH	FBLN1	FGF7	FIBIN	FKBP14	FMOD
FNDC1	FRMD6	GALNT1	GAS1	GEM	GJA1	GJB2
GLIS2	GLT8D2	GOLM1	GPC1	GXYLT2	HEG1	HES1
HSP90B1	HSPA1A	HSPA1B	IFI27	IGFBP3	IGFBP4	IGFL2
IKBIP	IL1R1	JUN	KDELR3	KLF10	LATS2	LMCD1
LOX	LOXL1	MANF	MCFD2	MDK	MFAP5	MINDY2
MIR4435-2HG	MMP14	MMP2	MPZL1	MXRA5	NBL1	NFYB
NNMT	NOTCH2	NR4A1	OSTC	P4HB	PDGFRL	PDLIM3
PDLIM4	PDPN	PEA15	PLAT	PLAU	PLIN3	PLOD2
PLPP3	PMP22	PPP2CB	PRSS23	PTGFRN	PTS	PTTG1IP

PXDN	RAB31	RCN1	RECQL	RTN4	S100A16	SDC1
SERPINE1	SERPINH1	SHC1	SLC39A6	SNX9	SPOCK1	SPON1
SRM	SULF1	SULF2	SUMO3	SURF4	THBS1	THY1
TIMP2	TIMP3	TMED2	TMEM158	TMEM263	TNFRSF12A	TNFRSF1A
TPBG	TUBB6	TWIST1	TXNDC5	UGDH	VAMP5	VIM
ZFP36L1	ZYX					

Table S4. The sequences for shRNA, related to STAR Methods.

**shRNA Target**	**Sequence (5' to 3')**
Notch1 (human)	shNotch1, GGAGCATGTGTAACATCAA
	shCtrl, TTCTCCGAACGTGTCACGT
PLAU (human)	shPLAU, CGCATGACTTTGACTGGAATT
	shCtrl, CCTAAGGTTAAGTCGCCCTCG
Plau (mouse)	shPLAU, CCCACTACTATGGCTCTGAAA
	shCtrl, CCTAAGGTTAAGTCGCCCTCG

Table S5. Primers for cloning and overexpression of target molecules, related to STAR Methods.

**Target**	**Vector/Plasmid**	**Primer Name**	**Primer Sequence (5' to 3')**
PLAU (human)	pHAGE-CMV vector	Forward	CGACGCGTGCCACCATGAGAGCCCTGCTGGCGCG
		Reverse	GCGTCGACGAGGGCCAGGCCATTCTCTTCCTTGG

Table S6. Primers for qRT-PCR, related to STAR Methods.

**Genes**	**Forward Primer Sequence (5' to 3')**	**Reverse Primer Sequence (5' to 3')**
TMED2(human)	GGTCTCGGGCTATTTCGTTAG	GCCACCTCGAAGATGAGGC
PLAU (human)	GGGAATGGTCACTTTTACCGAG	GGGCATGGTACGTTTGCTG
CAV2 (human)	AAGACCTGCCTAATGGTTCTGC	CTCGTACACAATGGAGCAATGAT
ANXA2 (human)	TCTACTGTTCACGAAATCCTGTG	AGTATAGGCTTTGACAGACCCAT
S100A16 (human)	ATGTCAGACTGCTACACGGAG	GTTCTTGACCAGGCTGTACTTAG
ANKH (human)	CATCACCAACATAGCCATCGAC	GCCAGCATCTCGACTGCAT
SURF4 (human)	ATGGGCCAGAACGACCTGA	ACTGGAACCACATACGGATGC
P21 (human)	TGTCCGTCAGAACCCATGC	AAAGTCGAAGTTCCATCGCTC
P53 (human)	CAGCACATGACGGAGGTTGT	TCATCCAAATACTCCACACGC
ACTB (human)	CATGTACGTTGCTATCCAGGC	CTCCTTAATGTCACGCACGAT
POSTN (human)	CTCATAGTCGTATCAGGGGTCG	ACACAGTCGTTTTCTGTCCAC
THBS1 (human)	TCACCACGTTGTTGTCAAGGG	AGACTCCGCATCGCAAAGG
MMP14 (human)	GATGGCCGCTGAGAGTGAC	GGCTACAGCAATATGGCTACC

SERPINE1 (human)	ACCGCAACGTGGTTTTCTCA	TTGAATCCCATAGCTGCTTGAAT
CCN2 (human)	AACCACGGTTTGGTCCTTGG	CAGCATGGACGTTCGTCTG
CXCL14 (human)	GTTCCAGGCGTTGTACCAC	CGCTACAGCGACGTGAAGAA
INHBA (human)	CCTCCCAAAGGATGTACCCAA	CTCTATCTCCACATACCCGTTCT
IGFBP3 (human)	GGTGATTCAGTGTGTCTTCCATT	AGAGCACAGATACCCAGAACT
IGFL2 (human)	AGAGAAGCCTGAGGAATTTACA AAAT	TGTCCCATCAGTCTCCCACAT
Notch1 (human)	GAGGCGTGGCAGACTATGC	CTTGTACTCCGTCAGCGTGA
Col4a1 (mouse)	CCTGGCACAAAAGGGACGA	ACGTGGCCGAGAATTTCACC
Hspg2 (mouse)	TTCCAGATGGTCTATTTCCGGG	CTTGGCACTTGCATCCTCC
Tnc (mouse)	TTTGCCCTCACTCCCGAAG	AGGGTCATGTTTAGCCCACTC
Mmp14 (mouse)	ACCCACACACAACGCTCAC	GCCTGTCACTTGTAAACCATAG A
Cxcl14 (mouse)	AGTGTAAGTGTTCCCGGAAGG	GCAGTGTGGGTACTTTGGCTT
Spp1 (mouse)	ATCTCACCATTCGGATGAGTCT	TGTAGGGACGATTGGAGTGAAA
Tgfb1 (mouse)	CCACCTGCAAGACCATCGAC	CTGGCGAGCCTTAGTTTGGAC
Cx3cl1 (mouse)	CTGGCCGCGTTCTTCCATT	GCACATGATTTCGCATTTCGT
Cdkn1a (mouse)	CGCAGGTTCTTGGTCACTGT	TGTTCACGAAAGCCAGAGCG
Trp53 (mouse)	CTCTCCCCCGCAAAAGAAAAA	CGGAACATCTCGAAGCGTTTA
Actb (mouse)	GGCTGTATTCCCCTCCATCG	CCAGTTGGTAACAATGCCATGT

Table S7. Details of clinical information of PDAC patients in-house cohort, related to STAR Methods.

**Patient ID**	**P1**	**P2**	**P3**
Cancer type	PDAC	PDAC	PDAC
Gender	Male	Female	Female
Age	53	64	68
Stage	IIB	IIA	IIB
Perineural invasion	Yes	Yes	Yes
Tumor grade	Intermediate	Intermediate	Poorly differentiated
Vascular invasion	Yes	No	Yes
Number of lymph nodes involved	1	0	2
Fibrosis	Moderate	Mild	Mild
